# dynamAedes: a unified modelling framework for invasive *Aedes* mosquitoes

**DOI:** 10.1186/s13071-022-05414-4

**Published:** 2022-11-08

**Authors:** Daniele Da Re, Wim Van Bortel, Friederike Reuss, Ruth Müller, Sebastien Boyer, Fabrizio Montarsi, Silvia Ciocchetta, Daniele Arnoldi, Giovanni Marini, Annapaola Rizzoli, Gregory L’Ambert, Guillaume Lacour, Constantianus J. M. Koenraadt, Sophie O. Vanwambeke, Matteo Marcantonio

**Affiliations:** 1 Georges Lemaître Center for Earth and Climate Research, Earth and Life Institute, UCLouvain, Louvain-la-Neuve, Belgium; 2 Unit Entomology and the Outbreak Research Team, Tropical Medicine Institute, Antwerp, Belgium; 3 Senckenberg Biodiversity and Climate Research Centre, Frankfurt am Main, Germany; 4 Institute of Occupational, Social and Environmental Medicine, Goethe University, Frankfurt am Main, Germany; 5 Medical and Veterinary Entomology Unit, Institute Pasteur du Cambodge, Phnom Penh, Cambodia; 6 Laboratory of Parasitology, National reference centre/OIE collaborating centre for diseases at the animal-human interface, Istituto Zooprofilattico Sperimentale delle Venezie, Legnaro, Italy; 7 The University of Queensland, School of Veterinary Science, Gatton, Australia; 8 Research and Innovation Centre, Fondazione Edmund Mach, San Michele all’Adige, Italy; 9 EID Méditerranée, Direction Technique, Montpellier, France; 10 Altopictus, Pérols, Mérignac, France; 11 Wageningen University & Research, Department of Plant Sciences, Laboratory of Entomology, Wageningen, The Netherlands; 12 Evolutionary Ecology and Genetics Group, Earth and Life Institute, UC Louvain, Louvain-la-Neuve, Belgium

**Keywords:** Biological invasions, Invasion ecology, Mosquitoes, Insects, Process-based models, Spatial epidemiology, Dispersal, Vector-borne pathogens

## Abstract

**Abstract:**

Mosquito species belonging to the genus *Aedes* have attracted the interest of scientists and public health officers because of their capacity to transmit viruses that affect humans. Some of these species were brought outside their native range by means of trade and tourism and then colonised new regions thanks to a unique combination of eco-physiological traits. Considering mosquito physiological and behavioural traits to understand and predict their population dynamics is thus a crucial step in developing strategies to mitigate the local densities of invasive *Aedes* populations. Here, we synthesised the life cycle of four invasive *Aedes* species (*Ae. aegypti*, *Ae. albopictus*, *Ae. japonicus* and *Ae. koreicus*) in a single multi-scale stochastic modelling framework which we coded in the R package dynamAedes. We designed a stage-based and time-discrete stochastic model driven by temperature, photo-period and inter-specific larval competition that can be applied to three different spatial scales: punctual, local and regional. These spatial scales consider different degrees of spatial complexity and data availability by accounting for both active and passive dispersal of mosquito species as well as for the heterogeneity of the input temperature data. Our overarching aim was to provide a flexible, open-source and user-friendly tool rooted in the most updated knowledge on the species’ biology which could be applied to the management of invasive *Aedes* populations as well as to more theoretical ecological inquiries.

**Graphical Abstract:**

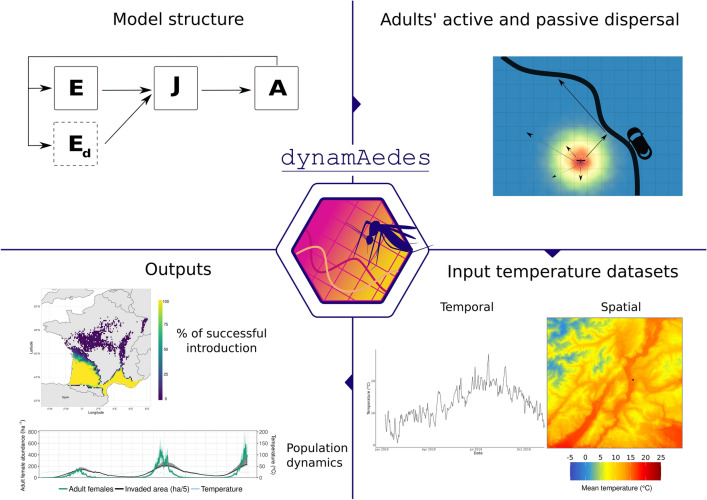

**Supplementary Information:**

The online version contains supplementary material available at 10.1186/s13071-022-05414-4.

## Background

Some mosquito species within the *Aedes* taxon have a unique combination of biological traits such as: (i) efficient transmission of viruses debilitating for humans and animals [1–3], (ii) eco-physiological plasticity that allows for rapid adaptation [4] and exploitation of novel environments created by humans [5] and (iii) egg stage with high resistance to dry and cold conditions which facilitate displacements over broad ecological and geographical distances [6–8]. Some of these species were accidentally brought outside their native areas by human activities and colonised new regions. These mosquitoes, often referred as “*Aedes* invasive mosquitoes” (AIMs), have attracted the interest of scientists and public health officers and much effort has been done to unravel their physiological and behavioural traits. Among these species, *Ae. aegypti*, *Ae. albopictus*, *Ae. japonicus* and *Ae. koreicus* showed an important expansion of their geographical ranges, with the first two species often causing burden on public health due to the viruses they transmit to humans. As a consequence, mechanistic models aimed to reproduce the basic life cycle of these four species have been developed starting from experimental and observational datasets on the relationship between environmental variables and physiological parameters (e.g. for *Ae. aegypti* [9–13]; for *Ae. albopictus* [14–18]; for *Ae. japonicus* [19]; for *Ae. koreicus* [20]). The inclusion of functions describing physiological and developmental rates into modelling frameworks allows for more reliable model extrapolations, as the chances of biologically unrealistic outcome may be lower compared to pure correlative model approaches [21, 22]. Comparisons between modelled and observed population trends showed that such mechanistic models can be used, for example, to understand population dynamics in space and time and thus can enhance the pest control strategies against AIMs [23].

Models targeting AIMs developed so far aimed to simulate the population dynamics of only one species at time and for a single qualitative (i.e. “individual”, “container” or “household”) or quantitative (cell in a lattice grid) spatial scale. Moreover, only a few of these models have been made readily operational, for example by organising them in open-access, user-friendly software or libraries with sufficient documentation for practical applications [24, 25]. SkeeterBuster, a container-level population dynamical model for *Ae. aegypti*, has been the first agent-based model for mosquitoes made available as a free (but not open-source) software [26]. Concerning *Ae. albopictus*, Erguler et al. [14] made available a model developed as a Python library that was afterwards wrapped into the R package albopictus. More recently, the European Centre for Disease Control (ECDC) has provided a free and open-source adaptation of a model initially developed by Tran et al. [17], by making it accessible via the R Shiny application AedesRisk.^1^ Similarly, two generic age and stage-structured discrete-time population dynamics models, also applicable to mosquitoes, were proposed in the last few years: stagePop ([27]; applied to *Ae. japonicus* in Wieser et al. [19] ) and sPop [28]. Despite the current availability of models applicable to invasive *Aedes*, none of them can be directly generalised across species while retaining biological credibility, e.g. species-specific models work only for a single species whereas generic models may oversimplify the life cycle structure or are not equipped with species-specific physiological parameters. Hence, if users decide to use generic models, they need to screen the scientific literature, filter and manipulate experimental data (often scarce and non-standardised) to inform models on the species of interest. Moreover, the available models often do not consider mosquito dispersal or even completely lack spatial structure.

Here, we aimed to fill these gaps by synthesising the life cycle of four AIMs species, *Ae. aegypti*, *Ae. albopictus*, *Ae. japonicus* and *Ae. koreicus*, in a single modelling framework, which we coded in the R package dynamAedes. dynamAedes integrates several eco-physiological and spatial aspects that were separately explored in previously published AIMs models (Additional file 1: Table S1). First, we designed a core model informed by temperature, larval density-dependent competition and photoperiod for four different *Aedes* species. These functions were calibrated by using state-of-the art knowledge on the thermal and dispersal biology of these species. Second, we accounted for the spatial and temporal heterogeneity of temperature estimates by defining three different spatial scales that users can explore: punctual, local and regional. These spatial scales were thought to meet different degrees of spatial complexity and data availability. Third, we integrated both active and human-mediated passive dispersal in the modelling framework, an aspect which has been rarely taken into account in previous studies aiming to model either AIMs habitat suitability or population dynamics. Finally, the whole dynamAedes project has an open-source and interdisciplinary rationale, which facilitates any potential future development by the scientific community.

Our overarching aim was to provide a flexible and open-source tool which can be used for applications to aid the management of invasive *Aedes* populations but also for more theoretical ecological inquiries. In this study, we described the model and applied it on four case studies, assessing the results by using observational mosquito data.

## Materials and methods

### A summary of invasive *Aedes* species ecology

#### *Aedes aegypti*

*Aedes (Stegomyia) aegypti* (Linnaeus, 1762), commonly referred to as the “yellow fever mosquito”, was progressively brought outside sub-Saharan Africa by human trade. It was first introduced in the Americas during the sixteenth century and afterwards to tropical and temperate regions of Asia and Oceania [29]. Its invasion was favoured by specific functional traits, such as egg desiccation resistance, which allowed them to withstand prolonged periods of dry conditions, or egg resistance to cold temperatures [7, 30, 31]. *Aedes aegypti* efficiently transmit several viruses to humans, including yellow fever, dengue, chikungunya, Zika, Rift Valley, Mayaro and eastern equine encephalitis viruses [32–34]. This “capacity” to transmit viruses is the results of several species-specific eco-evolutionary traits, such as: (i) very high preference for human hosts (anthropophily), which is channelled by genetic traits linked to behavioural and physiological evolutionary advantages [5, 35], (ii) widespread exploitation of human dwellings and architectures as shelter, hide and resting indoor sites (endophily) to avoid unfavourable environmental conditions [36, 37] and (iii) selection of artificial containers for oviposition and subsequent larval development (eusynantrophy; [38]).

#### Aedes albopictus

*Aedes (Stegomyia) albopictus* (Skuse, 1895), commonly referred as the “Asian tiger mosquito”, is native of tropical and subtropical regions of Southern-East Asia and Indonesia [39, 40]. It is a competent vector of several viruses, including dengue, chikungunya, Zika, West Nile, eastern equine encephalitis and La Crosse viruses [41–43] and it has been implicated as the vector species causing local transmission of dengue, chikungunya or Zika virus, also at temperate latitudes outside its native areas [44–50]. This species is a more opportunistic feeder compared to *Ae. aegypti* [51]. It prefers sub-urban habitats with the presence of vegetation, dispersing bites among several species, a behaviour that might decrease the likelihood of pathogen transmission to humans [52, 53]. Populations of this species located at temperate latitudes show: (i) adaptation to temperate climatic conditions [54] and (ii) a stronger tendency to laying diapausing eggs at the end of summer [55, 56]. Diapausing eggs were found to be resistant to below-freezing temperatures and allowed *Ae. albopictus* populations to overwinter and thrive at higher latitudes than *Ae. aegypti* [7, 55].

#### *Aedes japonicus japonicus*

*Aedes (Hulecoeteomyia) japonicus japonicus* (Theobald, 1901) [Hulecoeteomyia japonica], the “Asian bush mosquito”, originated in an area comprised between East China, East Russia and Japan [57]. This species may be competent for the transmission of pathogens of medical importance for humans, such as dengue, West Nile, Zika and Usutu viruses, but only experimental evidence of its role as vector exists ([58–67], but see [68] for an estimate of the risk of transmitting West Nile virus by this species). This species lesser role as a vector for human pathogens may also be derived from its tendency to feed often on other species than humans as well as the preference for more natural over urbanised areas. Established populations of *Ae. japonicus* have been detected in North America since 1998 and more recently also in European countries [64, 69–73]. This species is well adapted to cold climates where it overwinters either as larvae or as diapausing eggs [74] in areas where larval habitats freeze completely during winter [75, 76, 64].

#### *Aedes koreicus*

*Aedes (Hulecoeteomyia) koreicus* (Edwards, 1917) (Hulecoeteomyia koreica), commonly referred to as the “Korean bush mosquito”, is native to temperate areas of Northeast Asia comprising Russia, the Korean peninsula, Japan and Northeast China [77]. This species is a suspected vector of *Dirofilaria immitis*, Japanese encephalitis and chikungunya viruses, but it has not yet been directly implicated in transmission events of zoonotic pathogens [77–79]. *Aedes koreicus* is well adapted to temperate climates [8] and it has recently colonised areas of Central Europe, where it is in constant range expansion [8, 69, 79–86]. *Aedes koreicus* seems to prefer rural over highly urbanised habitats and has been found to feed on other species than humans [51, 87, 88]. In areas where *Ae. koreicus* lives in sympatry with other invasive *Aedes* species, the Korean bush mosquito is able to colonise higher altitudes and its development can start earlier in the season with respect to other AIMs [84]. This trait may give them a competitive advantage over other container-breeding mosquito species whose adults emerge later in the season.

### Theoretical structure of the model

dynamAedes is a multi-compartmental model where the survival and transition rates are defined as binomial draws informed by temperature, photoperiod and density-dependent functions. The basic structure of dynamAedes was described in Da Re et al. [10], which already included active and human-mediated passive dispersal for *Ae. aegypti*; however, here we amended some components of the model to generalise its structure to include three other AIMs. Thus, we provide here a short recap of core model structure while describing the new features of the model.

**Fig. 1:**
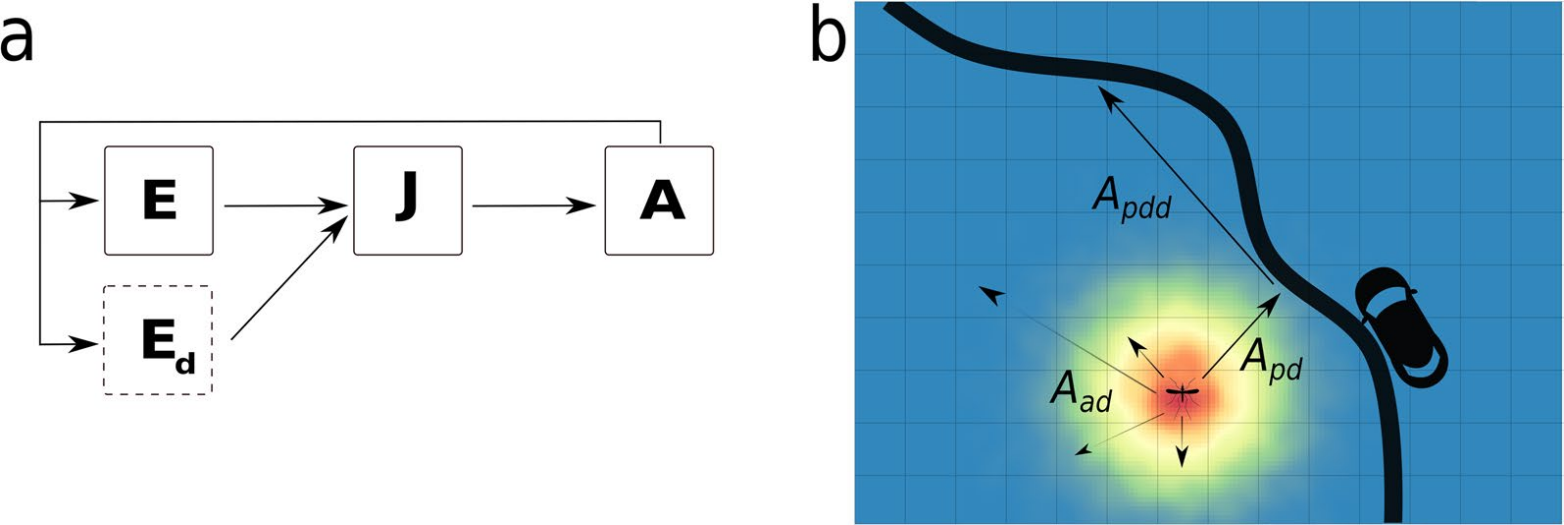
Graphical representation of dynamAedes model structure, adapted from Da Re et al. [10]: **a** the life cycle of a generic simulated mosquito species, while in **b** a representation of active and passive dispersal processes happening within the Adult (A) compartment at local scale. E: egg compartment; Ed: diapause egg compartment (available for all species except *Ae. aegypti*), *J* juvenile compartment, *A* adult compartment, *Aad* adult active dispersal, *Apdd* adult passive dispersal, *Apd* adult probability of getting into a car

dynamAedes is composed of three main compartments (life stages) that represent a simplified version of *Aedes* mosquito life cycle: egg, juvenile and adult stages (Fig. 1). Larval and pupal stages, which can be assumed to have somewhat similar physiological requirements, are fused in a unique “juvenile” compartment. Each compartment is divided into sub-compartments to account for the different physiological states for mosquitoes in the three main compartments (e.g. 1-day-old adult females that are not sexually mature). The number of sub-compartments in each compartment is dictated by the known minimum number of days that each species needs to pass to the next developmental stage or to complete the gonotrophic cycle (for adults). Thus, the minimum duration of development in each compartment varies among developmental stages as well as among species. As an example, the whole duration of the developmental cycle (i.e. from egg-laying to adult emergence) has a minimum duration of 11 days for *Ae. aegypti* and *Ae. albopictus*, whereas 21 days for *Ae. koreicus* and *Ae. japonicus* (see Additional file 1: Table S2).

In the model, “time” is treated as a discrete quantity and “day” is the fundamental temporal unit. Therefore, each event in the simulated life cycle occurs once per day and always in the same order. The model can be run with or without a spatial structure. If the model is spatially explicit, space is treated as a discrete quantity. In this case, the fundamental spatial unit is a (user-defined) cell of a lattice grid into which the species life cycle takes place and, if relevant (see below), among which adult mosquitoes disperse.

Adult female mosquitoes lay non-diapausing eggs, E, or diapausing eggs, Ed, according to geo-location and time of year (i.e. according to the photoperiod). Both the embryonic development and the hatching of diapausing eggs are activated by increasing daily temperature and photoperiod. All the developmental and reproductive events considered in the model were treated as stochastic processes with probabilities derived from temperature (or photoperiod)-dependent functions by following the generic formulation:

1 $$\begin{aligned} X^{event}_{s,t} \sim Binomial(X_{s,t-1},\pi _X) \end{aligned}$$

where $X_{s,t-1}$ may represent eggs, juveniles or adults that undergo one of the following events in the life cycle: lay eggs, hatch, emerge or survive in cell *s*, at the end of the day $t-1$. $\pi _X$ is the temperature-dependent (or photoperiod dependent for the hatching of diapausing eggs) daily probability of any of the life cycle events *X*. All the temperature-dependent functions were calibrated using data from the scientific literature (Additional file 1: Table S3) fitted using exponential or polynomial equations as well as non-linear Beta density functions, by using a combination of drc (non-linear models) and aomisc (Beta function self-starters) R packages [89, 90]. The Beta function derives from the Beta density function and it has been adapted to describe phenomena taking place only within a minimum and a maximum threshold value (threshold model), such as physiological rates dependent on temperature in the mosquito life cycle [89]. In a similar fashion, adult active dispersal was modelled as species-specific log-normal decaying functions of distances derived from dispersal estimates from field observations for *Ae. aegypti* and *Ae. albopictus* ([71, 91–93]; see Additional file 1: Table S4 for dispersal parameters). Besides active dispersal, the model also considers dispersal aided by cars along the main road network (a matrix containing the coordinates of the grid cells of the landscape intersecting the road network must be provided; see the “Spatial scales of the model and temperature data sources” section), defined as the probability of a female mosquito to get into a car, be driven and released further away along the road network. This probability has been defined for all species by dispersal data for *Ae. albopictus* [94], while the average distance covered by a single car trip per country was taken on Pasaoglu et al. [95]. This type of dispersal is thought to be amongst the main drivers of medium-range geographical expansion for AIMs [71, 84, 94].

**Fig. 2:**
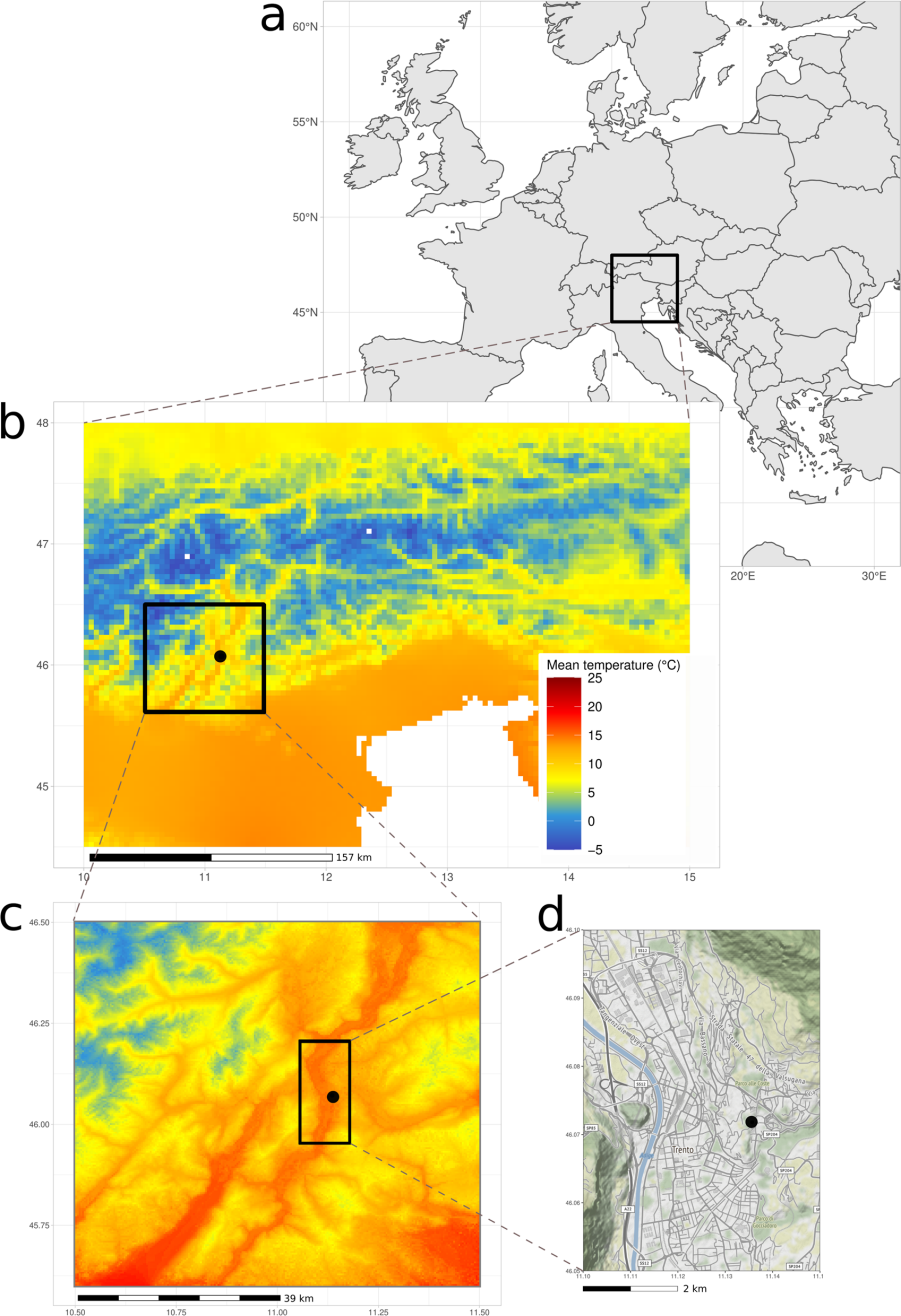
dynamAedes allows to simulate *Aedes* mosquitoes population dynamics at three different spatial scales. Considering the European continent as example (**a**), dynamAedes can predict the mosquito population dynamics at regional (**b**), local (**c**) and punctual (weather station; **d**) scale. Passive and active dispersal is enabled only at local spatial scale

Density-dependent survival is an important regulatory factor of mosquito population dynamics [96]. Its regulatory effect on juvenile stages appears to be more common in mosquitoes breeding in container or highly ephemeral habitats [97], such as invasive *Aedes* species. Unlike the first conceptualisation of the model presented in Da Re et al. [10], in dynamAedes we parameterised a density-dependent function by extracting observations on *Ae. aegypti* from Fig. 2a and b published in Hancock et al. [98] by using the Webplot Digitizer [99]. We considered the proportion of juveniles that survived through the juvenile stage (in a 2-l container) reported by these authors as an estimate of juvenile survival probability at different densities. Mortality probabilities ($1-proportion\;surviving$) were converted into rates, which were scaled to a daily time step dividing by the corresponding immature development time (in days) at different densities. Finally, we regressed the natural logarithms of these daily mortality rates on the corresponding densities. The fitted daily survival rate at different densities was then summed to the temperature-dependent juvenile mortality. The resulting probability was used to inform a binomial random draw (see Eq. 1) which defined the overall juvenile daily survival.

Some invasive *Aedes* can lay eggs resistant to low temperature that are commonly referred to as “diapausing eggs” [7, 56, 74]. Diapause describes an evolutionary adaptation of an insect species to overcome unfavourable environmental conditions by passing through an alternative and dormant physiological stage. In *Ae. albopictus*, maternal photoperiod is the environmental stimulus implied to induce oviposition of “diapausing eggs” [56, 100]. Diapause is a crucial biological process for *Ae. albopictus*, *Ae. japonicus* and *Ae. koreicus*; thus the oviposition of diapausing/non-diapausing eggs was integrated in dynamAedes as a species-specific exponential function describing the proportion of diapausing eggs given photoperiod length (and thus geographically dependent; [101, 102]). The function is based on data from Lacour et al. [56] for *Ae. albopictus* and Krupa et al. [74] for *Ae. japonicus*. We applied the same diapausing function developed for *Ae. japoncus* to *Ae. koreicus* because of the close phylogeny of these species and the lack of data for *Ae. koreicus* (Additional file 1: Table S5). The daily survival of diapausing eggs was set to be constant (0.99) only for *Ae. japonicus* and *Ae. koreicus*, while for *Ae. albopictus* we used the exponential function described in Metelmann et al. [15]. The hatching rate of diapause eggs was triggered by an increasing photoperiod regime (spring): beginning with 11.44 hours of light for *Ae. albopictus* (95th percentile estimated from Petrić et al. [101]) and 10.71 hours for *Ae. japonicus* or *Ae. koreicus* [74].

The set of core equations describing the compartmental model dynamics, dispersal kernel parameters and shapes of temperature-, photoperiod- and density-dependent functions is reported in the Additional file 1.

### Overview of the R package

The function dynamAedes.m calls the model and allows to customise the simulated population dynamics scenario through a suite of options. As for the simplest application of the model (no explicit spatial dimension, scale = "ws"; see next paragraph for further details), the user has to define what species to model through the argument species (default "aegypti"), “type” and number of introduced propagules through intro.eggs, intro.deggs (for diapausing eggs), intro.juvenile or intro.adults (default intro.eggs = 100, intro.deggs = 0, intro.juvenile = 0, intro.adults = 0) and the volume (L) of water habitats in each spatial unit with the argument jhwv (juvenile-habitat water volume, parameterised from Hancock et al. [98]; default jhwv = 2; Additional file 1: see Figure S7 for a sensitivity analysis of this and other key model parameters). Moreover, the argument temps.matrix takes the matrix of daily average temperature used to fit the life cycle rates. This matrix must be organised with the daily temperature observations as columns and the geographic position of the *i*-grid cell as rows (it follows that the matrix will have only one row when scale = "ws"). The first column in temps.matrix must match temporally the date of start of the simulations, which is set with startd using the “YYYY-MM-DD” date format (in the same way, the date of end of simulations can be set with endd), while the number of model iterations can be defined by the argument iter. The model has been optimised for parallel computing and the number of parallel processes can be specified through the argument n.clusters.

The default output of dynamAedes.m consists of a list of numerical matrices containing, for each iteration, the number of individuals in each life stage per day (and for each grid cell of the study area if scale = "lc" or "rg"). If the argument compressed.output = FALSE (default TRUE), the model returns the daily number of individuals in each life stage sub-compartment. The model, coded in the R statistical language [103], is available on the CRAN.

#### Spatial scales of the model and input temperature data

The selection of the geographical scale for population dynamics is a crucial aspect of the whole package and the temperature dataset provided to dynamAedes.m function must reflect this decision. Along with the photoperiod, temperature is the other environmental driver of our model; this choice was dictated by the important role of ambient temperature in the mosquito life cycle. The measurement of temperature is scale-dependent; thus we structured the model to allow for temperature datasets relevant for different spatial scales (Fig. 2) and to match the different hypotheses that users may want to test. The punctual or “weather station” scale (scale = "ws") is the smallest geographical scale (i.e., no spatial dimension) available in dynamAedes and the reference environment is assumed to be what represented by the location of the chosen weather station (or any other data-loggers). In this case, the model has no spatial structure, thus dispersal is not considered: the model will return the temporal trend of population dynamics given the chosen temperature, juvenile-habitat water volume and photoperiod conditions. The “local” scale (scale = "lc") represents scenarios and spatial resolutions at which species dispersal and local microclimate variability are relevant for the users. The spatial resolution of the matrix of temperatures should be kept equal or smaller than the maximum daily dispersal range of the mosquito species (i.e., usually < 1 km for *Aedes* species; [104]). The optional arguments cellsize, dispbins and maxadisp are available to fine tune the dispersal kernel, which drives the spatial behaviour of the simulated mosquito populations. The argument cellsize (default "cellsize = 250" m) sets the minimal distance of the dispersal kernel and should match the size of the cell to avoid inconsistencies (i.e. mosquitoes dispersing at a finer or courser spatial resolution than the matrix of temperatures), maxadisp sets the maximum daily dispersal (default maxadisp = 600 m) and dispbins the resolution of the dispersal kernel (default dispbins = 10). Passive dispersal is also implemented and it requires (i) a matrix containing the coordinates of the grid cells of the landscape intersecting the road network (argument road.dist.matrix) and (ii) to specify the average car trip distance through the argument country, which can be defined by the user or considering estimates for the following countries: France, Germany, Italy, Poland, Spain and the UK [95]. An extensive example of model application at local spatial scales is described in Da Re et al. [10]. The rationale behind the third spatial scale considered in the model, the “regional” scale (scale = "rg"), was to return an overview of *Aedes* population dynamics over large spatial extents (i.e., spatial resolution > 1 km). The regional scale model does not account for species dispersal, so that introductions happen separately (but at the same time) in each grid cell, which hence are closed systems. The output of the model at “regional” scale can be compared to those produced by correlative Species Distribution Models (SDMs), with the advantage of mechanistic rather than purely correlative model foundations. If the modelled species is *Ae. albopictus*, *Ae. japonicus* or *Ae. koreicus* (e.g. species = "albopictus"), at punctual scale the arguments defining latitude (lat) and longitude (long) should be adequately defined to allow a correct egg-diapausing dynamics. The amount of water available for larval development in each spatial unit(s) (at any model spatial scale) was set as 2 l, which is the water volume considered in the experiments we used to parametrise model functions [98]. It is likely that for many real-world model applications, the relative availability of breeding habitats is much higher, and we encourage users to set a value based on their scenarios and hypotheses (i.e., through the model option jhwv; [105]).

#### Auxiliary functions

Several auxiliary functions are available to analyse model outputs. The function psi returns the proportion of model iterations that resulted in an viable population for the given date. It works for all spatial scales and a output can reflect either the overall grid or each single cell. Likewise, summaries of mosquito abundance at each life stage for each day can be obtained through adci, which by default returns the inter-quartile range abundance of each life stage. Similarly, icci returns a summary of the number of invaded cells over model iterations estimates of the dispersal (in km$^{2}$) for the simulated mosquito populations is provided by the function dici, which is available only for model results computed at the local scale (the only scale which integrates dispersal).

## Case studies and model validation

We applied the model to four case studies representing different geographical scales and areas, species and invasion trajectories. The case studies were chosen considering the availability of mosquito datasets to show model strength and weakness. We did not report any example for the “local scale” as it had already been provided in Da Re et al. [10], who applied a previous version of dynamAedes.

### Regional-scale models

#### Likelihood of successful introductions of *Ae. albopictus* and *Ae. aegypti* across California, USA

We used dynamAedes at “regional” scale to assess the likelihood of successful introductions of two invasive *Aedes* species across California (USA): *Ae. aegypti* and *Ae. albopictus*, which are considered established from 2011 and 2013, respectively [106, 107]. California is among the few areas where established populations of these two species were detected during the last decade and their progressive spread was documented in great detail.^2^ We downloaded daily minimum and maximum temperature data from the NASA Daily Surface Weather Data on a 1-km Grid for North America (DAYMET), version 4 [108], from 1 January 2011 to 31 December 2018. These two sets of data (in netCDF format) were clipped to the boundary of California and aggregated at a spatial resolution of 2.5 km by using a combination of GDAL [109] and Climate Data Operators (CDO; [110]) software. The two sets of raster layers were then imported in R 4.0.4 [103], transformed in matrices, averaged and converted to integers to obtain a single average daily temperature integer matrix with cell id as observations (rows) and days as variables (columns). This dataset was used as the input temperature matrix for the model. We run 80 model iterations for 5 years, introducing 500 eggs in each cell of the gridded landscape on 15 May 2011 and 15 May 2013 for *Ae. aegypti* and *Ae. albopictus*, respectively. By using the auxiliary function psi, we then derived a map showing the proportion of iterations with viable population of both *Ae. aegypti* and *Ae. albopictus* at the end of the simulated period (15 May 2016 and 2018). The amount of breeding habitat was set to 100 l (per cell), which we assumed to be representative of the potential water larval habitats available given the cell size and the overall regional climate.

We validated model prediction using maps of cities in California with known species presence updated to 2021 by the California Department of Public Health (CDPH)^3^, We derived the average predicted percentage of successful introduction for each city and computed the area under the ROC curve (AUC) score, which defines the probability that a randomly chosen positive city will be ranked higher than a randomly chosen negative city. An AUC score $> 0.5$ indicates that the model is performing better than random, while a score of 1 indicates perfect prediction. In addition, we calculated the percentage of positive cities that fell into a grid cell that had a probability of establishment ≥ 1% (e.g. at least 1 out of the $\hbox {n}_{{th}}$ iterations reported a viable mosquito population in that cell), defining this metric as Sensitivity1%.

**Fig. 3:**
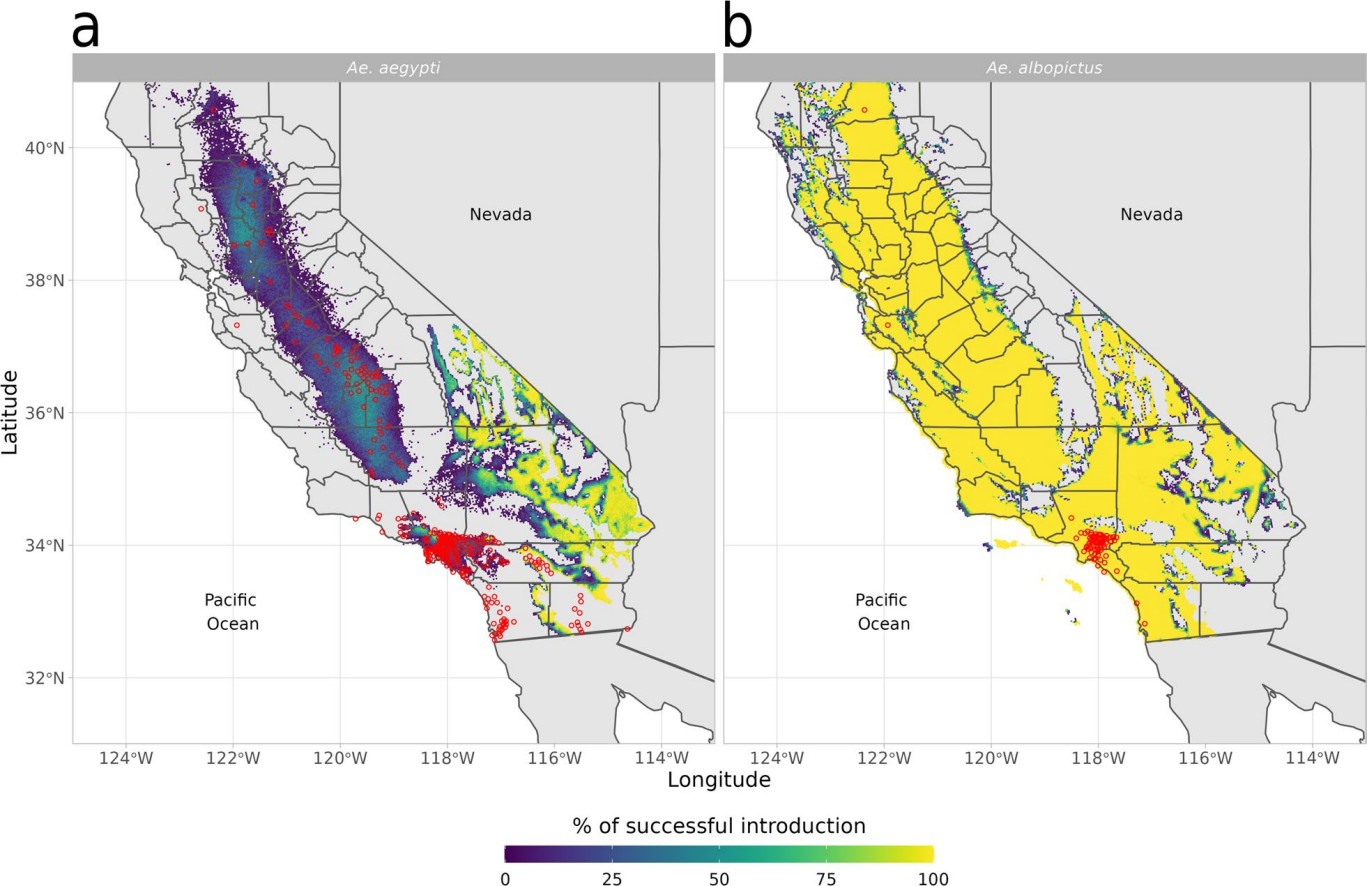
Predicted percentage of established introductions of *Ae. aegypti* **a** and *Ae. albopictus* **b** in California (USA) for the years 2011–2016 and 2013–2018, respectively. The red dots represent the centroids of the Californian municipalities with established populations in 2021 as reported by the Californian Department of Public Health (CDPH)

The predicted spatial pattern of areas with a high likelihood of *Ae. aegypti* and *Ae. albopictus* successful introduction is in general consistent with updated information on the presence of this species (Fig. 3). Predictions show percentage of a successful introduction above zero in all counties with known presence of these species, except for the extreme south-east coastal part of the state, where *Ae. aegypti* was predicted to have a zero or low percentage of successful introductions whereas it is well established (Fig. 3). This may be due to the micro-climatic variability that characterises coastal areas of California, which may not be resolved by the temperature datasets that we have considered in this case study. Contrarily, areas predicted to be suitable for *Ae. albopictus* exceeded by far the known current distribution of this species. Factors other than temperature and photoperiod, more nuanced aspects of the species invasion history and the extremely low humidity during the dry season in the Central Valley of California or the Inland Empire may hinder species establishment in these areas. Nevertheless, *Ae. albopictus* was recently found as far north as Redding, Shasta County; thus it is not unlikely that this species is also present (perhaps at low densities), but not yet detected, along the central part of the Central Valley of California.

Both models had > 75% successful introduction scores (Sensitivity1%) when validated at city level (Table 1). Still, only the prediction for *Ae. aegypti* validated at city level reported an AUC score > 0.5 (Table 1), whereas validating the model by averaging the predictions at county level resulted in an AUC > 0.5 for both species (0.903 [0.814–0.992] and 0.560 [0.277–0.843] for *Ae. aegypti* and *Ae. albopictus*, respectively; Additional file 1: Figure S8).

#### Likelihood of successful *Aedes albopictus* introductions in France

*Aedes albopictus* was first detected in metropolitan France in 1999 [111] and since 2004 it has established populations in the southern part of the country while still expanding its distribution range [69]. We used dynamAedes model at “regional” scale to assess the success of introductions of *Ae. albopictus* for the whole of metropolitan France. We processed ERA5-Land [112] hourly air temperature measured at 2 m above surface from 1 January 2015 to 31 December 2020 in the Climate Data Store (CDS) Toolbox^4^ to get the daily mean temperature of the period considered for the whole of metropolitan France, at the spatial resolution of $\sim$ 9 km. The netCDF file obtained was imported in R 4.0.4 [103], where it was clipped to the extent of metropolitan France, converted from degrees Kelvin to degrees Celsius and then to integer to obtain a single average daily temperature integer matrix with cell id as observations (rows) and days as variables (columns). This dataset was then used as the input temperature matrix for the model. We ran 100 simulations for 5 years, introducing 500 eggs in each cell of the gridded landscape on 15 May 2015. By using the auxiliary function psi, we derived a map of the areas showing the proportions of iterations that produced a viable population of *Ae. albopictus* 5 years after the simulated introductions.

**Fig. 4:**
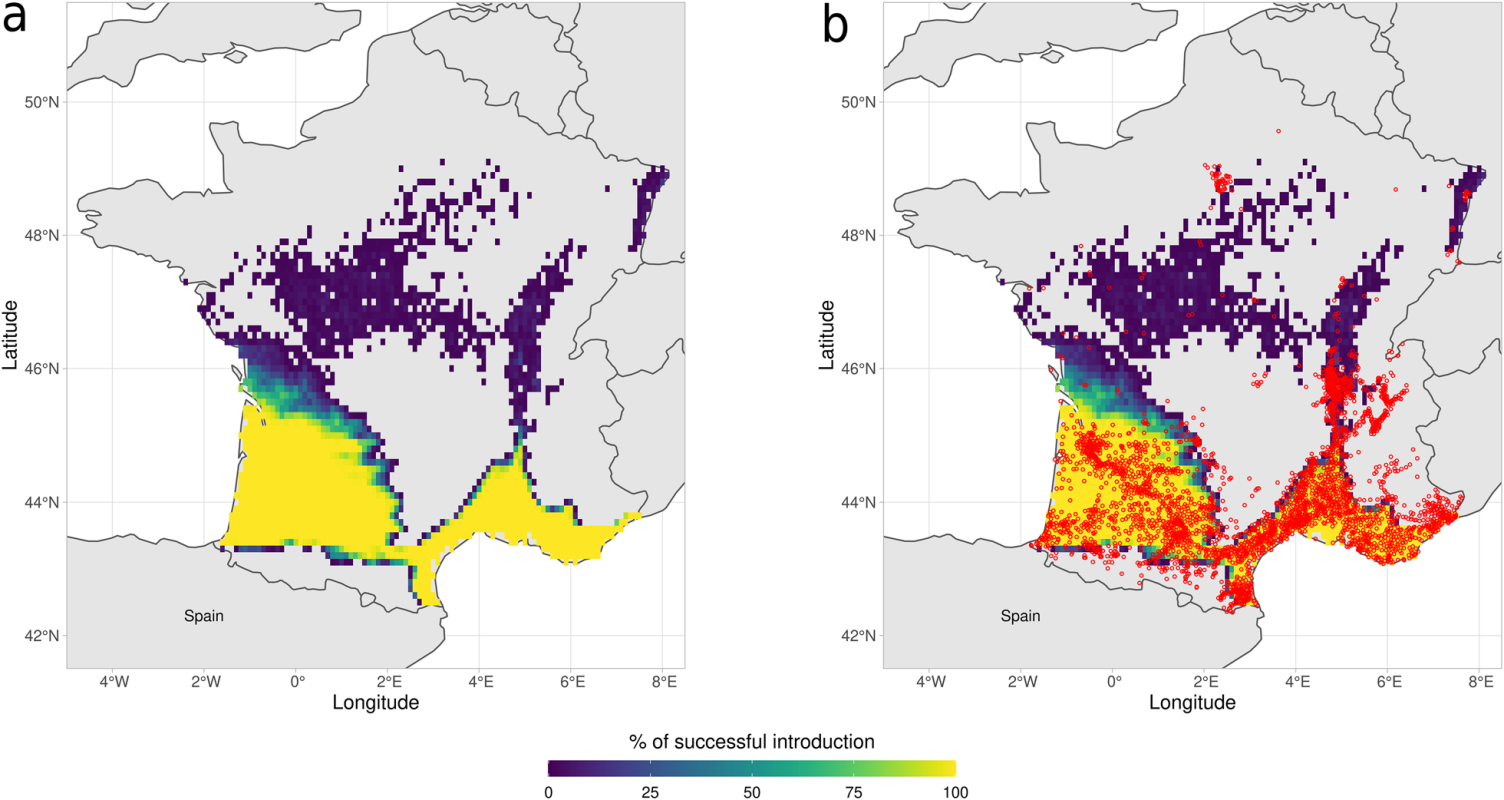
Percentage of successful introduction for *Ae. albopictus* in France for the years 2015–2020: **a** Model prediction, **b** model prediction and, in red dots, the centroids of the French Municipalities with established population of *Ae. albopictus* reported by the French Health Ministry (SI-LAV)

We validate the model predictions using the list of the 3419 French municipality reporting established *Ae. albopictus* populations provided by the French Health Ministry (data referring to 2020)^5^. Similarly to the previous case study, we computed both the AUC as well as the proportion of species occurrence locations falling into a grid cell that had a percentage of established introductions ≥ 1%. The spatial pattern of the areas predicted to have a high likelihood of successful *Ae. albopictus* introduction (Fig. 4) is consistent with updated observational data (*Ae. albopictus* map; ECDC, 2020) as well as with the results of other mechanistic models (see for instance [15, 16]). The Mediterranean French coast and the Rhone valley are the areas where our model predicted the highest percentage of successful introduction. Similarly, the Aquitaine region on the Atlantic coast and the Alsace region in the north-east part of France showed relatively high predicted percentage of successful introduction. The northern and the central parts of France, as well as the Pyrenees areas, show low percentage of successful introductions under current climatic conditions. However, the resolution of the pixel, may have influenced model outcome especially in topographically complex areas such as the Pyrenees or the French Alps, where the microclimate of the valleys may be masked. Similarly, the model was not able to predict the successful introduction of the species in areas such as the metropolitan zone of Paris, where *Ae. albopictus* is established and favoured by urban heat island effect and the continuous inflow of species propagules from areas where the species is well established. Most railways, flights and highways have a connection with Paris; for example the Paris-Lyon-Mediterranée axis is the main transportation artery of France, with an average of $> 60,000$ cars/day on the highway^6^ and 240 trains per day.^7^ The high propagule pressure in Paris may compensate for the less favorable climatic conditions which drove our simulated introductions to extinction (recent phylogeographic findings support this hypothesis; see Sherpa et al. [113]). Overall, all the considered model performance metrics indicate the capacity of dynamAedes to discriminate between areas where AIMs species can or cannot establish viable populations (Table 1).

### Punctual population dynamics temporal trends

#### *Aedes albopictus* population dynamics in Nice, South-East France

*Aedes albopictus* is established mainly in the southern part of metropolitan France where it has colonized > 40% of municipalities^8^ and it is still expanding. We computed the population dynamics of the species by informing dynamAedes at “punctual” spatial scale by using temperature observations downloaded from the National Oceanic and Atmospheric Administration (NOAA) network via the R package rnooa. The observations of the NOAA weather station located in Nice area (usaf code = 076900, Lat 43$^{\circ }$ 42’00”N, Lon 7$^{\circ }$ 13’12” E, 3.7 m a.s.l.), spanning from 1 January 2013 to 31 December 2018, were linearly interpolated to fill missing values. Afterwards, the daily average for all the observations was computed. We ran 100 simulations for 5 years, introducing 500 eggs on 15 May 2013. We then derived the daily abundance inter-quantile range for each life stage and the abundance of newly laid eggs per day by using the auxiliary function adci. The model was validated comparing the simulated eggs laid per day to egg counts from mosquito ovitrap data, following the validation approach presented in Tran et al. [17]. During the years 2014–2018, 50 ovitraps were installed in the Nice area and inspected fortnightly from April–May to November–December (data kindly provided by EID Méditerranée). We computed the Spearman’s *rho* correlation coefficient between the weekly-aggregated simulated newly-laid eggs and the mean observed eggs per day.

**Fig. 5:**
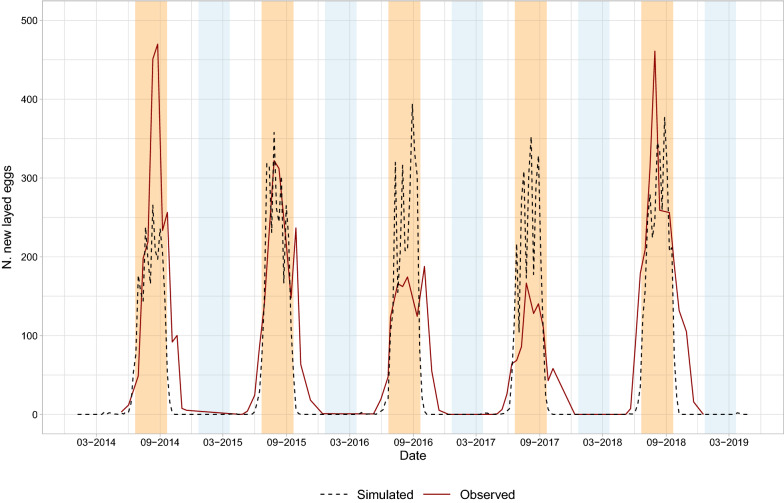
Temporal trend reporting simulated (dashed black line) and observed (red line) new-laid eggs of *Ae. albopictus* in Nice, SE France (2014–2018). The light blue bands represent the winter seasons, whereas the orange bands summer seasons. The simulated data were rescaled for graphical purposes by using the ratio between the maximum observed value, and the maximum median simulated value

Results showed that the model was able to correctly reproduce the seasonal dynamics of the new laid eggs over 5 years (Spearman’s *rho* = 0.753,* p* value $< 0.001$). The first simulated eggs were laid during the late spring of each year, confirming the fact that the first overwintering eggs hatch at the end of the winter season or at the beginning of spring. Field data indicated that oviposition generally ended in late autumn, whereas our model predicted a slightly shorter oviposition period. Nevertheless, the model was able to correctly infer the peak of the ovipositing season during the summer months (Fig. 5).

**Fig. 6:**
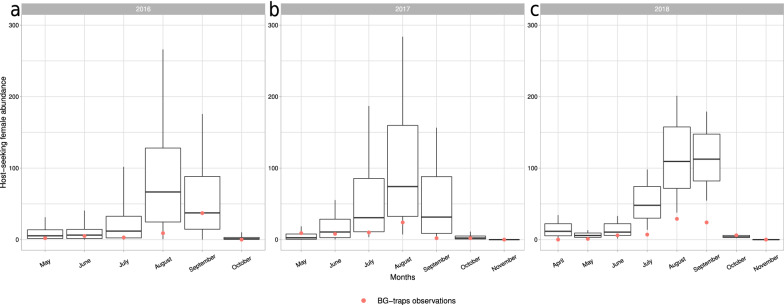
The distributions of simulated population abundances (boxplots) and the relative observed median abundance (red dots) of *Ae. koreicus* host-seeking females for the years 2016 (**a**), 2017 (**b**) and 2018 (**c**) in Trento, NE Italy

#### *Aedes koreicus* population dynamics in Trento, North-East Italy

*Aedes koreicus* was first detected in the Autonomous Province of Trento (NE Italy) in 2013, soon after the first detection in Italy (in the neighbouring Belluno province [81]).

We computed the population dynamics of the species by performing dynamAedes model at “punctual” spatial scale by using temperature observations downloaded from the local network of weather stations.^9^ The daily average temperature observations from the “Trento Laste” weather station (lat 46$^{\circ }$ 04’18.5” N, Lon 11$^{\circ }$ 08’08.5” E, 312 m a.s.l.) spanning from 1 January 2015 to 31 December 2018 were linearly interpolated to fill in missing values. We ran 100 simulations for 5 years, introducing 500 eggs on 15 May 2015. Using the auxiliary function adci, we then derived the daily inter-quartile range abundance for each life stage and for the daily host-seeking female sub-compartment (Fig. 6).

The model was validated computing Spearman’s *rho* correlation coefficient between the monthly-aggregated simulated host-seeking females and the observations gathered from four BG-Sentinel traps installed in Trento municipality from April to November 2016–2018 (data obtained from Marini et al. [20]). To compare observed and simulated data, the whole simulated host-seeking females abundance was multiplied by a BG-sentinel catching rate equal to 0.157, as estimated by Marini et al. [20] and similar to what was already reported for *Ae. albopictus* in previous studies [114].

The simulated population dynamics showed that *Ae. koreicus* can successfully establish and reproduce in the study area. The model correctly predicted the start of population dynamics in early spring, whereas the higher abundance of female host-seeking mosquitoes was predicted to be in late summer. Overall, our model was able to reproduce the observed seasonal population dynamics: 76.2% (47.6%) of the observed data were within the 95% (50%) credible intervals of model predictions (Additional file 1: Table S6). Similarly, the Spearman’s *rho* when considering the aggregated 3-year period was 0.735 (*p* value $< 0.001$) (Table 1).

**Table 1:** Summary table reporting the metrics used for validating the simulated population dynamics in each of the case study

Species	Geographic location	Scale	AUC	Specificity	Sensitivity	Sensitivity 1%	Spearman’s $\rho$
*Ae. aegypti*	California	Regional	0.658 (0.401-0.914)	0.8	0.642	0.795	–
*Ae. albopictus*	California	Regional	0.464 (0.402-0.527)	0.184	0.756	1	–
*Ae. albopictus*	France	Regional	0.842 (0.834-0.85)	0.817	0.783	0.723	–
*Ae. albopictus*	S-E France	Punctual	–	–	–	–	0.753***
*Ae. koreicus*	N-E Italy	Punctual	–	–	–	–	0.735***

The column “Sensitivity 1%” reports the proportion of cities predicted to have at least one successful introduction over the total number of iterations (predicted introduction success ≥ 1%. ***p value $< 0.001$)

## Discussion

By using dynamAedes, we were able to simulate with good accuracy the population dynamics of different AIMs species at different geographic scales and locations. The model showed overall good validation performance, and areas predicted to support *Aedes* mosquito populations matched observations or results from other models [115–117].

Nevertheless, good quality data on temperature-dependent survival and developmental rates are scarce for *Ae. koreicus* and *Ae. japonicus*. Similarly, mosquito observational datasets with sufficient longitudinal depth are rare for *Ae. koreicus* and presently absent, to the best of our knowledge, for *Ae. japonicus*. Thus, whilst we have built the foundations for an open-source modelling framework that can be progressively expanded, life cycle functions and model outputs of these two species may presently be less accurate than those for *Ae. aegypti *and *Ae. albopictus*.

Our modelling approach focused on the “species fundamental thermal niche” concept (*sensu* Hutchinson [118]) by considering temperature as the main driver of population dynamics. In light of this, the choice of temperature datasets is crucial [119]; for example, pixel size may influence model outcome because of the aggregating effect of the Modifiable Unit Areal Problem [120–122]. Climatic reanalysis and global/regional circulation models are reliable data sources with high temporal resolution for present climatic conditions and robust future projections, but they have coarse spatial resolution that may underestimate the microclimate and its effects on species biology [15, 123]. Coarse resolution of input temperature data may invalidate results in topographically complex regions where the effect of microclimatic variation on population dynamics may pass undetected. For example, the results showed in Figs. 3, 4 may be the consequence of coarse temperature input data, which caused a lower proportion of established populations in topographically complex areas such as the Pyrenees (France) or the Peninsular Ranges (California). Interpolated local micro-climatic conditions, for example estimated with the microclima R package [124], have the advantage of providing fine spatial and temporal resolutions datasets. However, they need to be properly validated with field data which are difficult to gather also over small geographical extents. Thus, temperatures measured with weather stations may be considered as the most accurate available observations of local climatic conditions. Moreover, though the availability of weather stations is limited and data often present temporal gaps, they may be more suited for statistical downscaling or bias adjustement for climate change projections [125].

### Model assumptions

We designed dynamAedes to be as ecologically relevant as possible by considering the most updated available biological data. However, data on mosquito development, survival and dispersal rates were sometimes limited and thus we relied on a set of “expert-based” assumptions that we report below.

The interplay of multiple environmental factors drives the population dynamics of *Aedes* mosquitoes, but we chose to base our model just on established information available for temperature, larval density-dependent competition and photoperiod [100, 126, 127]. This choice was suggested by a generalised lack of clear relationships between other environmental factors and *Aedes* population dynamics. For example, concerning the role of precipitations, different studies report contrasting results [9, 17, 128]. Moreover, AIMs mostly thrive in urban or suburban landscapes where the presence of standing water is often independent from precipitations (except for extreme rainfall events) [129]. At the present stage, dynamAedes is better suited for applications in temperate climates, where temperature seasonality is a more important limiting factor than in tropical climates, where other factors may limit mosquito life cycle [130].

We considered the effect of intra-specific competition on larval survival (but not development) by generalising the information available for *Ae. aegypti* to the other *Aedes* species due to a lack of species-specific experiments [98]. We did not consider other biotic interactions such as prey-predator dynamics or food and space competition with other mosquito taxa during the larval stages, although these factors influence the trajectory of introduced populations [88, 131–135].

Finally, we did not consider evolutionary processes which may affect invasion success over medium-long time spans. Given the reproductive strategy of *Aedes* mosquitoes, rapid evolutionary processes may take place over relatively short temporal periods (e.g. decades), allowing introduced populations to colonise areas outside their original ecological niche [5].

### Proposed research directions

dynamAedes is an open-source tool for testing ecological hypothesis and to support management plans concerning AIMs. The selection of areas at risk of AIMs establishment or periods when AIMs abundances are more likely to peak should be considered as important facets of AIMs surveillance. The importance of such early information becomes fundamental for protecting human health when dealing with AIMs that transmit pathogens, given the rapid ongoing climate changes. Mosquitoes are affected by temperature changes in often predictable ways. Changes in population dynamics can be extremely rapid and models can help tracking them. Modelling population dynamics under climate change scenarios is therefore important to anticipate AIMs population changes in space and time and thus to help mitigate potential human health risks.

The conceptualisation and design of dynamAedes required the review of up-to-date ecological and physiological scientific literature available on four *Aedes* species, which was integrated with feedback from expert ecologists and medical entomologists. From this process we learned that while information on some species can sometimes be abundant, the overall available data are poor, highly fragmented (e.g. Cebrián-Camisón et al. [51]) and often dependant on experimental settings (e.g. Kramer et al. [31]). For example, there is a large (but fragmented) body of data collected on *Ae. aegypti* and *Ae. albopictus* physiological rates [126], whereas datasets on *Ae. japonicus* and *Ae. koreicus* are just starting to be collected (i.e., *Ae. koreicus*: [136]; *Ae. japonicus*: [19, 64, 76]), and much work remains to be done on these species. Information regarding dispersal is scarce and available only for *Ae. aegypti* and *Ae. albopictus*. Passive dispersal through auto-vehicles was estimated only for *Ae. albopictus* [94], though the worldwide spread of AIMs was most likely caused by means of passive dispersal. We suggest that a standardised review effort together with the creation of centralised data repositories would help the exploitation of existing datasets for process-based models and hence to define which eco-physiological traits are understudied and should be targeted experimentally. This process has already been carried out with great success in other scientific disciplines such as plant functional ecology [137] or systems biology [138].

We believe that a closer interaction between modellers and experimenters will motivate the collection of standardised data on eco-physiological AIMs rates that will lead to more accurate model predictions. This project was inspired by such interactions and, in this spirit, dynamAedes was meant to be modified to relax its assumptions and limitations with new available information for anyone having basic R programming skills.

## Conclusion

In this study, we presented dynamAedes, a mechanistic process-based model, to infer invasive *Aedes* mosquito spatio-temporal population dynamics. This first version of the model was reliable in terms of both biological realism and statistical accuracy. The open-source nature, as well as the accessibility and flexibility of the project programming language offer great potential to further develop the model when new experimental observations will become available. Simulated mosquito abundance from dynamAedes could be used to inform epidemiological models (e.g., SIR or SEIR), and thus used to obtain risk estimates of mosquito-borne pathogen transmission. Finally, it is not unrealistic to extend the model to other invasive species in the genus *Aedes*, such as *Ae. notoscriptus*, to species of medical interest belonging to other genera in the *Culicidae* family or even to other non-mosquito blood-sucking insects such as *Culicoides*.

## Supplementary Information

**Additional file 1: Figure S1**. Overview of the temperature-dependent functions used in the model for the four *Aedes* species.** Figure S2**. Overview of the temperature-dependent functions used in the model for *Ae. aegypti*.** Figure S3**. Overview of the temperature-dependent functions used in the model for *Ae. albopictus*.** Figure S4**. Overview of the temperature-dependent functions used in the model for *Ae. japonicus*.** Figure S5**. Overview of the temperature-dependent functions used in the model for *Ae. koreicus*.** Figure S6**. Overview of the photoperiod-dependent diapause function used to in the model for *Ae. albopictus* and *Ae. japonicus*. The *Ae. japonicus* function was used for*Ae. koreicus* as well.** Figure S7**. Sensitivity analysis on the effect of (A) the variability of introduced propagules and juvenile-habitat water volume on the percentage of successful introduction; (B) the variability of the juvenile-habitat water volume on the median individual abundance.** Figure S8**. Predicted percentage of establishment of *Ae. aegypti*, *Ae. albopictus* in California (USA) for the years 2011–2016 and 2013–2018, respectively. Only pixels having a probability of successful introduction > 0 are shown. The red dots represent the counties where the species have been found. ** Table S1**. Description of mechanistic models for invasive *Aedes* available as software or scripts (online or on request).** Table S2**. Other model features. ** Table S3**. Species-specific temperature-dependent physiological parameters.** Table S4**. Species-specific dispersal parameters.**Table S5**. Species-specific photoperiod parameters.** Table S6**. Validation for *Ae. koreicus* model in Trento (NE Italy).

## Data Availability

The proposed methodology is free and open-source, and it is presently available at https://github.com/mattmar/dynamAedes/tree/master.

## Footnotes

1 AedesRisk v1.0: https://shinyapps.ecdc.europa.eu/shiny/AedesRisk/.

2 See https://maps.vectorsurv.org/.

3 California Department of Public Health, “Map and City List of *Aedes aegypti* and *Aedes albopictus* Mosquitoes in CA, 2011-2021”, accessed on 28 October 2021): https://www.cdph.ca.gov/Programs/CID/DCDC/Pages/VBDS.aspx.

4 CDS Toolbox:https://cds.climate.copernicus.eu/toolbox/doc/index.html.

5 https://signalement-moustique.anses.fr/signalement_albopictus/.

6 https://www.data.gouv.fr/fr/datasets/trafic-moyen-journalier-annuel-sur-le-reseau-routier-national/.

7 https://www.leparisien.fr/economie/l-europe-fait-passer-la-lgv-paris-lyon-a-l-heure-2-0-26-04-2018-7685634.php.

8 https://solidarites-sante.gouv.fr/sante-et-environnement/risques-microbiologiques-physiques-et-chimiques/especes-nuisibles-et-parasites/article/cartes-de-presence-du-moustique-tigre-aedes-albopictus-en-france-metropolitaine.

9 www.meteotrentino.it.
