# Supplementary material for: dynamAedes: a unified modelling framework for invasive Aedes mosquitoes
Source: Parasit Vectors. 2022 Nov 8;15:414. doi: 10.1186/s13071-022-05414-4 (PMC9641901; doi:10.1186/s13071-022-05414-4)
Supplement: Supplementary file 1 — Additional file 1: Figure S1. Overview of the temperature-dependent functions used in the model for the four Aedes species. Figure S2. Overview of the temperature-dependent functions used in the model for Ae. aegypti. Figure S3. Overview of the temperature-dependent functions used in the model for Ae. albopictus. Figure S4. Overview of the temperature-dependent functions used in the model for Ae. japonicus. Figure S5. Overview of the temperature-dependent functions used in the model for Ae. koreicus. Figure S6. Overview of the photoperiod-dependent diapause function used to in the model for Ae. albopictus and Ae. japonicus. The Ae. japonicus function was used forAe. koreicus as well. Figure S7. Sensitivity analysis on the effect of (A) the variability of introduced propagules and juvenile-habitat water volume on the percentage of successful introduction; (B) the variability of the juvenile-habitat water volume on the median individual abundance. Figure S8. Predicted percentage of establishment of Ae. aegypti, Ae. albopictus in California (USA) for the years 2011–2016 and 2013–2018, respectively. Only pixels having a probability of successful introduction > 0 are shown. The red dots represent the counties where the species have been found. Table S1. Description of mechanistic models for invasive Aedes available as software or scripts (online or on request). Table S2. Other model features. Table S3. Species-specific temperature-dependent physiological parameters. Table S4. Species-specific dispersal parameters.Table S5. Species-specific photoperiod parameters. Table S6. Validation for Ae. koreicus model in Trento (NE Italy). [file 13071_2022_5414_MOESM1_ESM.pdf]

Supplementary Materials
`dynamAedes`: a unified modelling framework for invasive *Aedes* mosquitoes

Daniele Da Re[1,*], Wim Van Bortel[2], Friederike Reuss[3,4], Ruth Müller[2], Sebastien Boyer[5], Fabrizio Montarsi[6] , Silvia Ciocchetta[7], Daniele Arnoldi[8], Giovanni Marini[8], Annapaola Rizzoli[8], Gregory L'Ambert[9], Guillaume Lacour[10], Constantianus J.M. Koenraadt[11], Sophie O. Vanwambeke[1], Matteo Marcantonio[12,*]

14th August 2022

[1] Georges Lemaître Center for Earth and Climate Research, Earth and Life Institute, UCLouvain, Louvain-la-Neuve, Belgium

[2] Unit Entomology and the Outbreak Research Team, Tropical Medicine Institute, Antwerp, Belgium

[3] Senckenberg Biodiversity and Climate Research Centre, Frankfurt am Main, Germany

[4] Institute of Occupational, Social and Environmental Medicine, Goethe University, Frankfurt am Main, Germany

[5] Medical and Veterinary Entomology Unit, Institute Pasteur du Cambodge, Cambodia

[6] Laboratory of Parasitology, National reference centre/OIE collaborating centre for diseases at the animal-human interface, Istituto Zooprofilattico Sperimentale delle Venezie, Legnaro, Italy

[7] The University of Queensland, School of Veterinary Science, Gatton, Australia

[8]Research and Innovation Centre, Fondazione Edmund Mach, San Michele all'Adige, Trento, Italy

[9] EID Méditerranée, Direction Technique, Montpellier, France

[10] Altopictus, Pérols, France

[11] Wageningen University & Research, Department of Plant Sciences, Laboratory of Entomology, Wageningen, The Netherlands.
[12] Evolutionary Ecology and Genetics Group, Earth and Life Institute, UCLouvain, Louvain-la-Neuve, Belgium

* corresponding author: daniele.dare@uclouvain.be; matteo.marcantonio@uclouvain.be

Contents

1 Review of mechanistic model for invasive *Aedes* **4**

2 Parameters used in `dynamAedes` **6**

3 Model core equations **12**

4 *Aedes sp.* response curve **14**

5 Juvenile-habitat water volume parameter sensitivity **20**

6 *Aedes aegypti* and *Ae. albopictus* regional scale case study **21**

7 *Aedes koreicus* population dynamics punctual scale case study **22**

1 Review of mechanistic model for invasive *Aedes*

Name	Year	Parameterized for (species)	Dispersal	Programming language	Reference
CIMSiM	1993	*Ae. aegypti*	No	Visual Basic	Focks et al. (1993b,a)
SkeeterBuster	2009	*Ae. aegypti*	No	C++	Magori et al. (2009)
`stagePop`	2009	Generic	No	R	Kettle and Nutter (2015)
	2011	*Ae. albopictus*	No	C++	Poletti et al. (2011)
ALBORUN	2013, 2019	*Ae. albopictus*	No	Ocelet	Tran et al. (2013, 2020)
`sPop`	2018	Generic	No	C++, R, Python	**?**
`albopictus`	2019	*Ae. albopictus*	No	C++, R, Python	Erguler et al. (2016, 2017)
	2019	*Ae. albopictus*	No	Octave v4.2.1, Runge–Kutta 4	Metelmann et al. (2019)
	2019	*Ae. koreicus*	No	R	Marini et al. (2019b)
ARBOCARTO	2019	*Ae. albopictus*, *Ae. aegypti*	No	Ocelet	Tran and Demarchi (2019)
	2020	*Ae. aegypti*	No	R	Iwamura et al. (2020)
	2020	*Ae. koreicus*	No	R	Kurucz et al. (2020)
	2020	*Ae. albopictus*	No		Pasquali et al. (2020)
	2021	*Ae. aegypti*	Yes	R	Da Re et al. (2021)
ALBOMAURICE	2021	*Ae. albopictus*	No	Ocelet	Iyaloo et al. (2021)
	2022	*Ae. japonicus*	Yes	R	Kerkow et al. (2022)
dynamAedes	2022	*Ae. aegypti*, *Ae. albopictus*, *Ae. japonicus*, *Ae. koreicus*	Yes	R	This study

Table 1: Description of mechanistic models for invasive *Aedes* available as software or scripts (online or on request).

2 Parameters used in `dynamAedes`

Species	Stage	Parameters	Main data source	Origin	Value	Comments
Ae. aegypti and Ae. albopictus	Eggs	min number of days for eggs development	Christophers (1960); Delatte et al. (2008)	Literature therein; La Reunion (France)	*4*	
Ae. aegypti and Ae. albopictus	Juveniles	min number of days for immature development	Christophers (1960); Delatte et al. (2008)	Literature therein; La Reunion (France)	*6*	
Ae. aegypti and Ae. albopictus	Adults	min number of days for gonotrophic cycle	Christophers (1960); Delatte et al. (2008)	Literature therein; La Reunion (France)	*4*	
Ae. koreicus and Ae. japonicus	Eggs	min number of days for eggs development	Dr Daniele Arnoldi unpublished results for *Ae. koreicus*	Trento (NE Italy)	8	
Ae. koreicus and Ae. japonicus	Juveniles	min number of days for immature development	Dr Daniele Arnoldi unpublished results for Ae. koreicus; Wieser et al. (2019)	Trento (NE Italy), Biberach (SW Germany)	9	min 10 days for *Ae. koreicus*, min 8 days for *Ae. japonicus*. Assumed 9 for both species
Ae. koreicus and Ae. japonicus	Adults	min number of days for gonotrophic cycle	Assumed the same of *Ae. aegypti*	-	4	Since the probability of complete the gonothropic cycle is roughly 1/3 of *Ae. aegypti*, we kept the same leght the same lenght of *Ae. aegypti*
All species	Juveniles	Survival rate (density-dependent)	Hancock et al. (2016)	Tropical (NE Australia)	Log-normal distribution	1-density dependant survival rate is additive to 1-temperature-dependent survial rate; the estimates made for *Ae. aegypti* were extended to the other three species
	Adults	Sex ratio 1:1	-	-	-	General modelling assumption widely taken in process-based modelling literature

Table 2: Other model features

Species	Stage	Model parameter	Data source	Origin	Function	Comments
Ae. aegypti	Egg	Survival rate	Thomas et al. (2012); Eisen et al. (2014)	Laboratory colony(Tropical Asia origin); Literature review	Beta	Data for T>0 taken from Eisen et al. (2014), data for T<0 taken from Thomas et al. (2012)
		Hatching rate	Christophers (1960); Farnesi et al. (2009); Mohammed and Chadee (2011)	Literature therein; Rock-feller strain; Trinidad and Tobago	Beta	48h estimates from Mohammed and Chadee (2011), divided by two to get a daily rate. Additional data: 0 hatching a 7°C from Christophers (1960) and 0.025 at 12°C daily hatching from Farnesi et al. (2009)
	Juvenile	Survival rate (temperature-dependent)	Yang et al. (2009)	NW of São Paulo State (Brazil)	Beta	Table 8 from Yang et al. (2009) Appendix1
		Emergence rate	Yang et al. (2009); Grech et al. (2015)	NW of São Paulo State (Brazil); Cordoba (Argentina)	Beta	Data from Yang et al. (2009) refer to all immature stages, we corrected them to match only pupa using information present in Grech et al. (2015) by taking the ratio between the minimum pupation time of the two experiment as a scaling factor
	Adult	Survival rate	Yang et al. (2009)	NW of São Paulo State (Brazil)	Beta	Table 4
		Gonotrophic cycle	Yang et al. (2009)	NW of São Paulo State (Brazil)	Beta	Table 5
		Oviposition: number of eggs	Christophers (1960); Yang et al. (2009)	Literature therein; NW of São Paulo State (Brazil)	Beta	Table 5 from Yang et al. (2009) rescaled using the average number of eggs/female reported in Christophers (1960).
Ae. albopictus	Egg	Survival rate (non-diapausing)	Metelmann et al. (2019); Expert based	Literature therein	nonlinear	Polynomial function taken from the SM manually adapted to get no survival at T>40°C
		Survival rate (diapausing)	Metelmann et al. (2019)	Literature therein	nonlinear	Polynomial function taken from the SM
		Hatching rate ((non-diapausing)	Delatte et al. (2008)	La Reunion (France)	Beta	Table 1; column Egg-L1
		Hatching rate (diapausing)	-	-	-	Assumed the same as for non-diapausing eggs
	Juvenile	Survival rate (temperature-dependent)	Metelmann et al. (2019)	Literature therein	nonlinear	Polynomial function taken from the SM
		Emergence rate	Delatte et al. (2008)	La Reunion (France)	Beta	Table 1; column Pupae-adult
	Adult	Survival rate	Metelmann et al. (2019)	Literature therein	nonlinear	Polynomial function taken from the SM
		Gonotrophic cycle	Delatte et al. (2008)	La Reunion (France)	Beta	Table 6

Species	Stage	Model parameter	Data source	Origin	Function	Comments
Ae. japonicus	Egg	Oviposition: number of eggs	Delatte et al. (2008)	La Reunion (France)	Beta	Table 6
		Survival rate	Wieser et al. (2019)	Biberach (SW Germany)	Beta	Observation taken from Fig. 1 in Wieser et al. (2019)
		Hatch rate	Wieser et al. (2019)	Biberach (SW Germany)	Beta	Reuss et al., unpublished. Hatching observed also below a thin ice layer (T<0)
	Juvenile	Survival rate	Wieser et al. (2019)	Biberach (SW Germany)	Beta	Reuss et al., unpublished
		Emergence rate	Reuss et al. (2018) Suppl. Mat.	Biberach (SW Germany)	Beta	Table S2; survival upper temperature limit was adapted by accounting for expert opinion.
	Adult	Survival rate	Reuss et al. (2018) Suppl. Mat.	Biberach (SW Germany)	Beta	Fig. 2
		Gonotrophic cycle	-	-	Beta	No information available, assumed the same of Ae. koreicus due to their phylogenetic and biogeographic similarity
		Oviposition: number of eggs	Reuss et al. (2018)	Biberach (SW Germany)	Beta	Number of eggs estimated by mean female wing length, using the formula taken from Armistead et al. (2008), their Fig. 5; we divided the estimated eggs per female per gonotrophic cycle by a factor of two, due the two ovipositing days we are accounting in the model.
Ae. koreicus	Egg	Survival rate	Marini et al. (2019b); Expert Based	Trento (NE Italy)	nonlinear	Polynomial function F3 and parameters values taken from Tab. 5 in Marini et al. (2019b) adjusted using observations provided in Arnoldi et al., unpublished observations
		Hatching rate	Marini et al. (2019b); Expert Based	Trento (NE Italy)	Beta	Table 1 scaled with unpublished data to account for non-embryonated eggs; Arnoldi et al., unpublished observations
	Juvenile	Survival rate (temperature-dependent)	Marini et al. (2019b); Expert Based	Trento (NE Italy)	Beta	Table 1 averaged from instar 1 to 4; the the upper limit has been adapted accordingly to expert opinions by Ciocchetta et al., unpublished observations and Arnoldi et al., unpublished observations
		Emergence rate	Marini et al. (2019b)	Trento (NE Italy)	Beta	Table 2
	Adult	Survival rate	Marini et al. (2019b); Expert Based	Trento (NE Italy)	Beta	Table 3
		Gonotrophic cycle	Marini et al. (2019b)	Trento (NE Italy)	Beta	Table 3
		Oviposition: number of eggs	Expert Based	Trento (NE Italy)	Beta	Arnoldi et al., unpublished observations

Table 3: Species-specific temperature-dependent physiological parameters

Species	Stage	Parameters	Main source	Origin	Distribution	Comments
Ae. aegypti	Adults	Active dispersal	Marcantonio et al. (2019)	California (USA)	Log-normal	Table 3
Ae. albopictus	Adults	Active dispersal	Marini et al. (2019a)	Rome (Italy)	Log-normal	Figure 2a; Data from the 3 MRR were fit with several distributions, best distribution chosen using AIC
Ae. japonicus	Adults	Active dispersal	-	-	Log-normal	No information available, assumed the same of *Ae. albopictus*
Ae. koreicus	Adults	Active dispersal	-	-	Log-normal	No information available, assumed the same of *Ae. albopictus*
	All species	Passive dispersal (average trip distance)	Pasaoglu et al. (2012)	-	-	Trip distance weighted average for ITA, DEU, FRA, ESP, POL, UK. Data taken from Figure 4.4 Average trip distance (km) by trip purpose using WebPlotDigitalizer
All species	Adults	Passive dispersal (Hitch-hiking probability)	Eritja et al. (2017)	Mediterranean (Catalonia, Spain)	gamma distribution	0.0051 probability of a female mosquito to enter a car; Assumed to be the same for all the species as estimated by Eritja et al. (2017) for *Ae. albopictus*

Table 4: Species specific dispersal parameters

Species	Stage	Model parameter	Data source	Origin	Function	Comments
Ae. aegypti	Diapause Egg	Diapause incidence	Lacour et al. (2015)	Ovitraps (Provence, Southern France)	Exponential	
Ae. japonicus	Diapause Egg	Diapause incidence	Krupa et al. (2021)	Ovitraps (Bas-Rhin, Northeast France)	Exponential	
Ae. koreicus	Diapause Egg	Diapause incidence	Krupa et al. (2021)	Ovitraps (Bas-Rhin, Northeast France)	Exponential	We used the same exponential function used for *Ae. japonicus* due to the close philogenetic relationship between these two species

Table 5: Species specific photoperiod parameters

3 Model core equations

The core set of equations that determine the size and dynamics of egg (E), juvenile (J) and adult (F) compartments in cell s, day t is:

$$E_{s,t} = E_{s,t}^{laid} + E_{s,t}^{survive} - E_{s,t}^{hatch} \tag{1}$$
$$J_{s,t} = E_{s,t}^{hatch} + J_{s,t}^{survive} - J_{s,t}^{emerge} \tag{2}$$
$$F_{s,t} = \frac{J_{s,t}^{emerge}}{2} + F_{s,t}^{survive} \tag{3}$$

The components of equations 2-4 can be synthetically described as:

$$E_{s,t}^{laid} \sim \sum_{F_{s,t}^{lay}} Poisson(\boldsymbol{R_E}) \tag{4}$$

Where $\boldsymbol{R_E}$ is a vector of number of eggs of length s in day t derived from a temperature-dependent function. $F_{s,t}^{lay}$ correspond to adult females which have matured eggs and can lay eggs for 2 days in a row.

$$E_{s,t}^{survive} \sim Binomial(E_{s,t-1}, \boldsymbol{\Pi_{Es}}) \tag{5}$$

Where $\boldsymbol{\Pi_{Es}}$ is a vector of length s containing the probabilities of egg survival in day t derived from a temperature-dependent function.

$$E_{s,t}^{hatch} \sim Binomial(E_{s,t-1}^{survive}, \boldsymbol{\Pi_{Eh}}) \tag{6}$$

Where $\boldsymbol{\Pi_{Eh}}$ is a vector of length s containing the probabilities of egg hatching in day t derived from a temperature-dependent function.

$$J_{s,t}^{survive} \sim Multinomial(J_{s,t-1}, \boldsymbol{\Pi_{Js}}) \tag{7}$$

Where $\boldsymbol{\Pi_{Js}}$ is a vector of length s containing the probabilities of juvenile survival in day t derived from the combination of temperature-dependent and density-dependent functions.

$$J_{s,t}^{emerge} \sim Multinomial(J_{s,t-1}, \boldsymbol{\Pi_{Je}}) \tag{8}$$

Where $\boldsymbol{\Pi_{Je}}$ is a vector of length s containing the probabilities of adult emergence in day t derived from a temperature-dependent function.

$$F_{s,t}^{survive} \sim Binomial(F_{s,t-1}, \boldsymbol{\Pi_{Fs}}) \tag{9}$$

Where $\boldsymbol{\Pi_{Fs}}$ is a vector of length s containing the probabilities of adult survival in day t derived from a temperature-dependent function.

4 *Aedes sp.* response curve

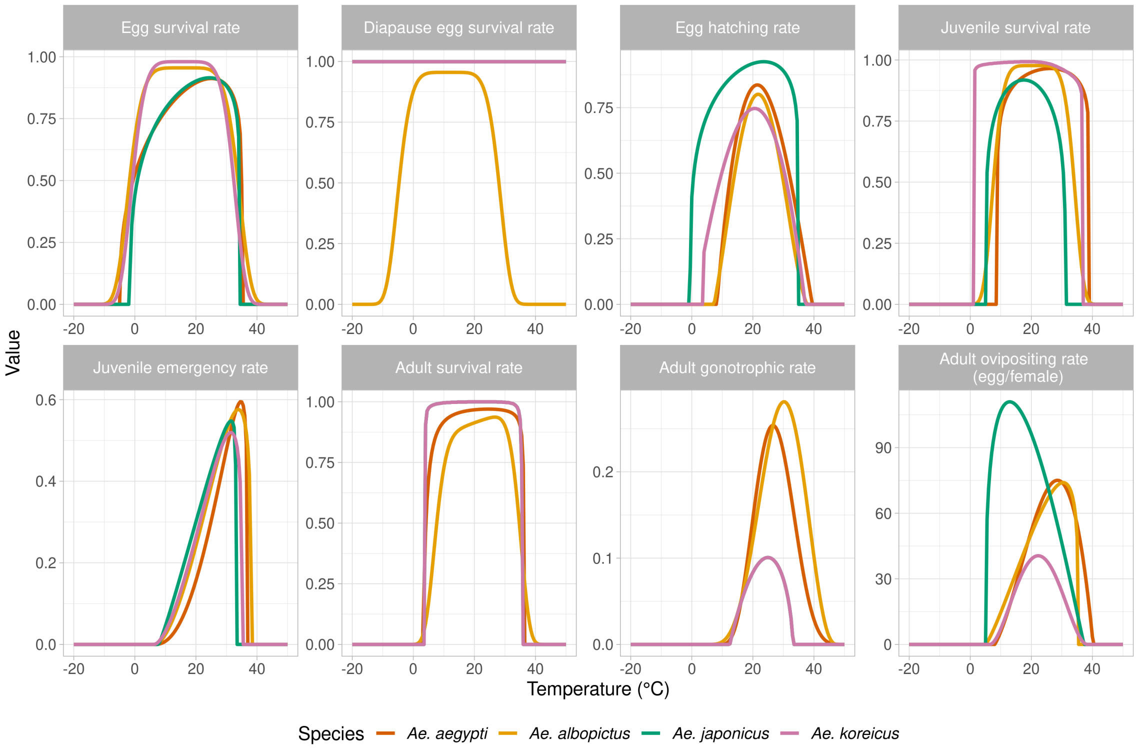

Figure 1: Overview of the temperature-dependent functions used in the model for the four *Aedes species*

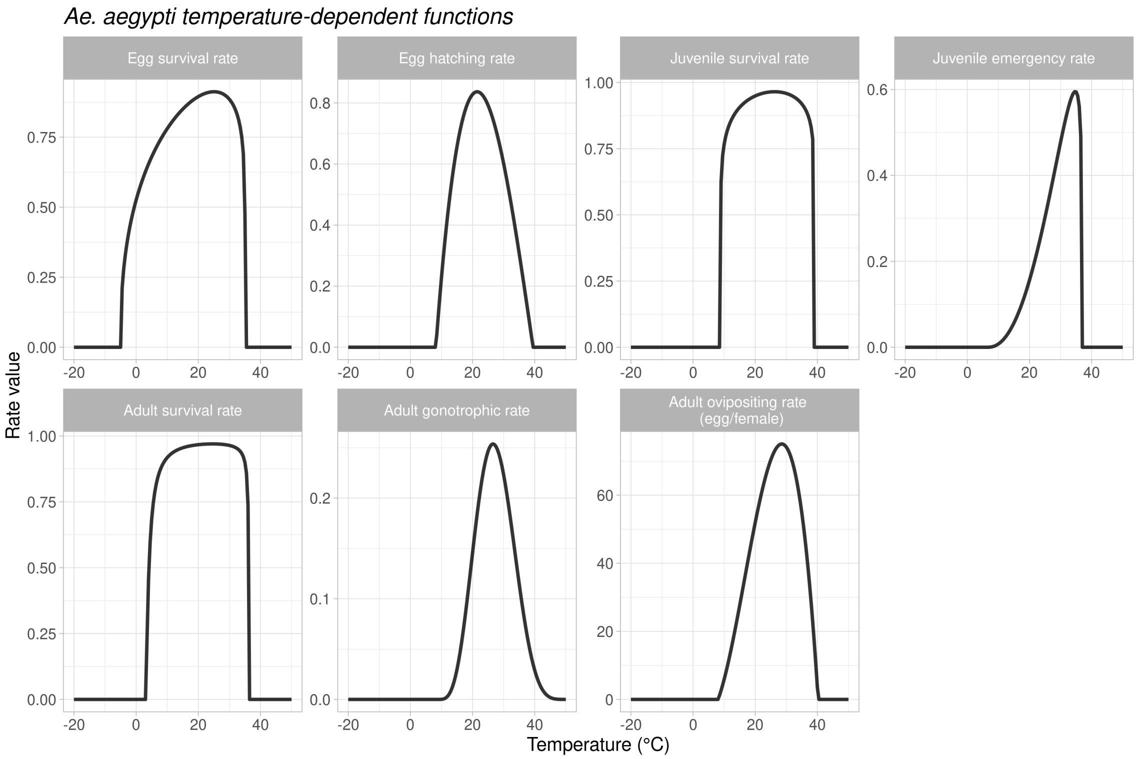

Figure 2: Overview of the temperature-dependent functions used in the model for *Ae. aegypti*

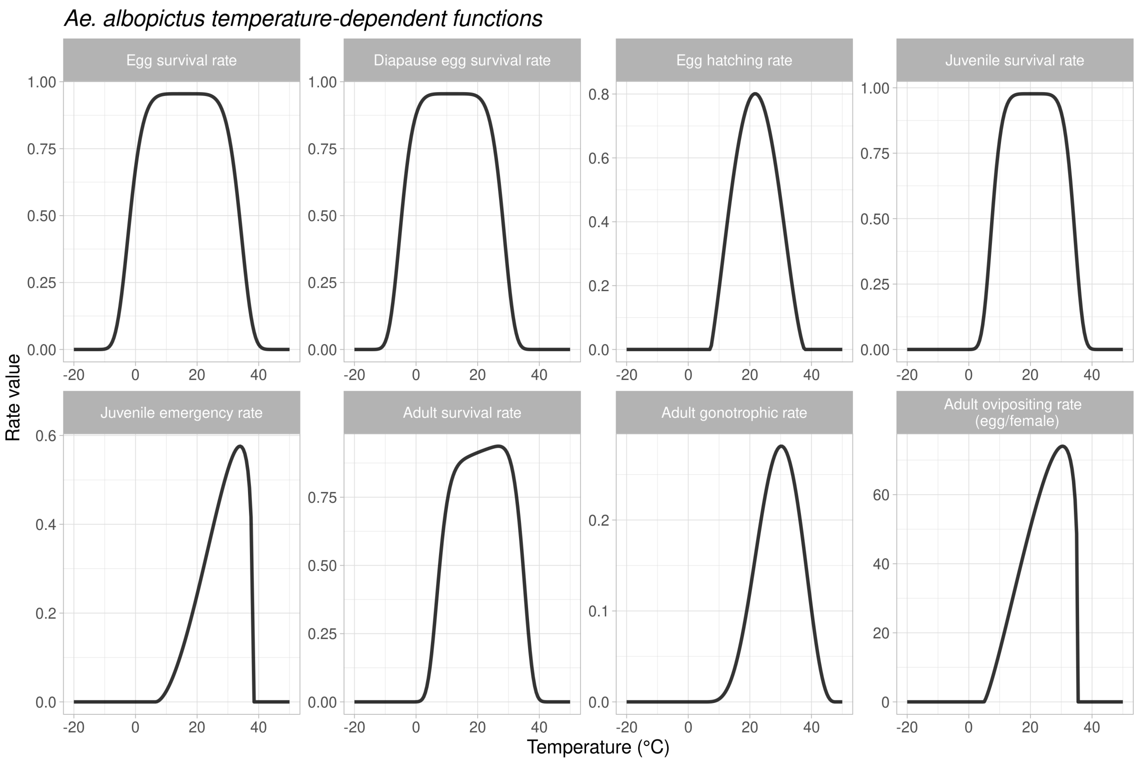

Figure 3: Overview of the temperature-dependent functions used in the model for *Ae. albopictus*

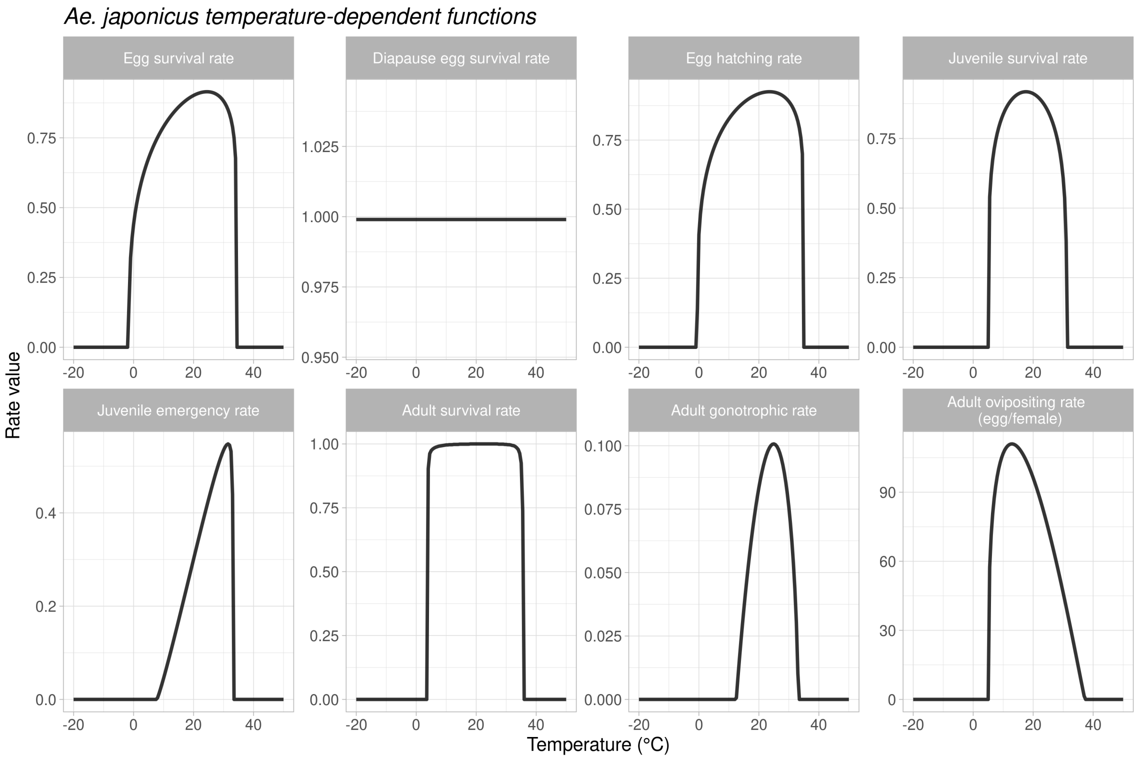

Figure 4: Overview of the temperature-dependent functions used in the model for *Ae. japonicus*

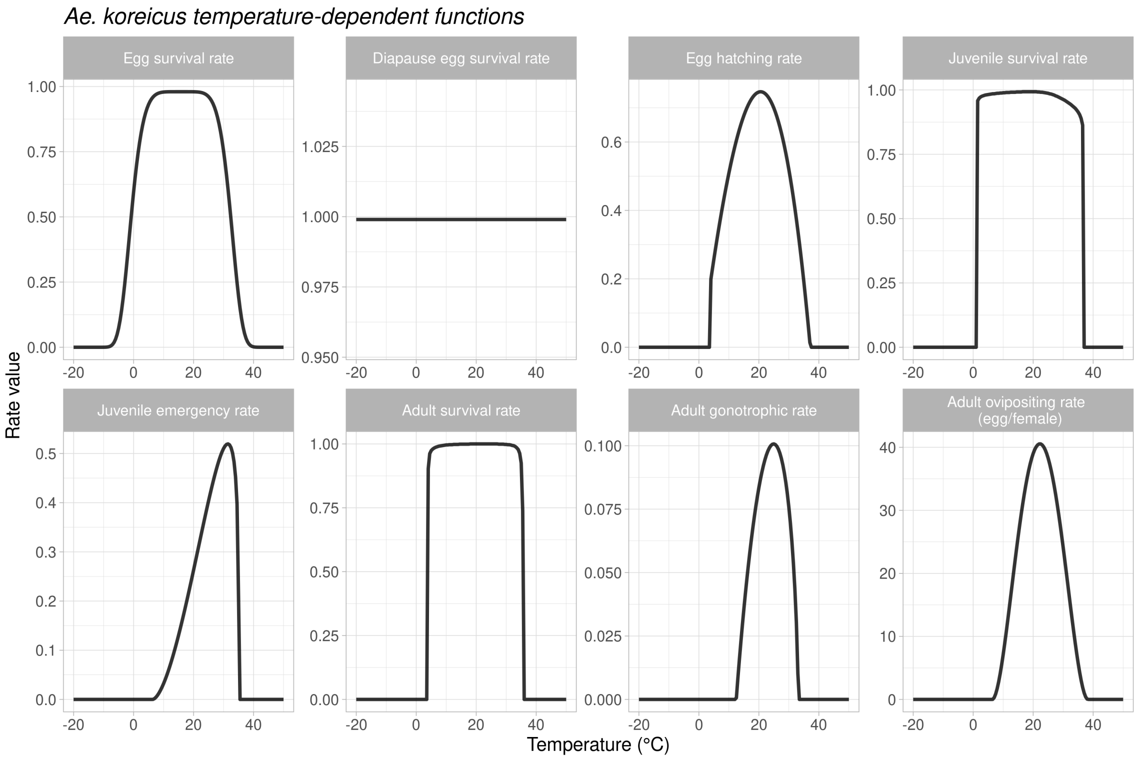

Figure 5: Overview of the temperature-dependent functions used in the model for *Ae. koreicus*

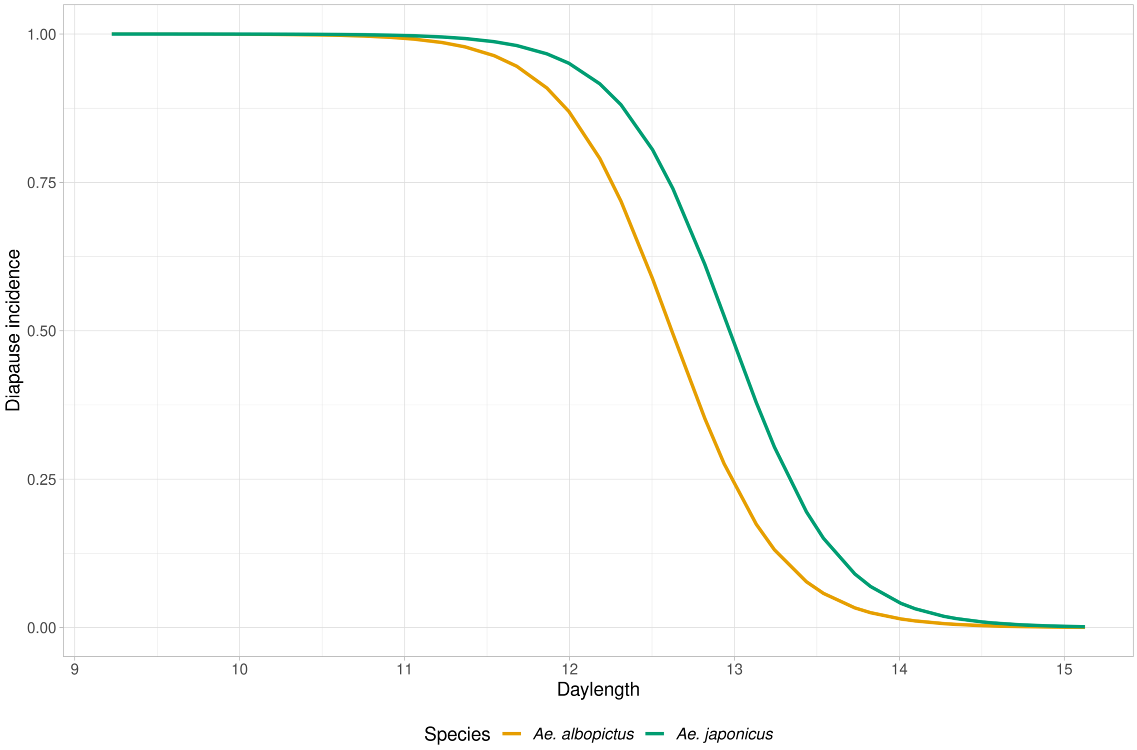

Figure 6: Overview of the photoperiod-dependent diapause incidence function used to in the model for *Ae. albopictus* and *Ae. japonicus*. The *Ae. japonicus* function was used for *Ae. koreicus* as well.

5 Juvenile-habitat water volume parameter sensitivity

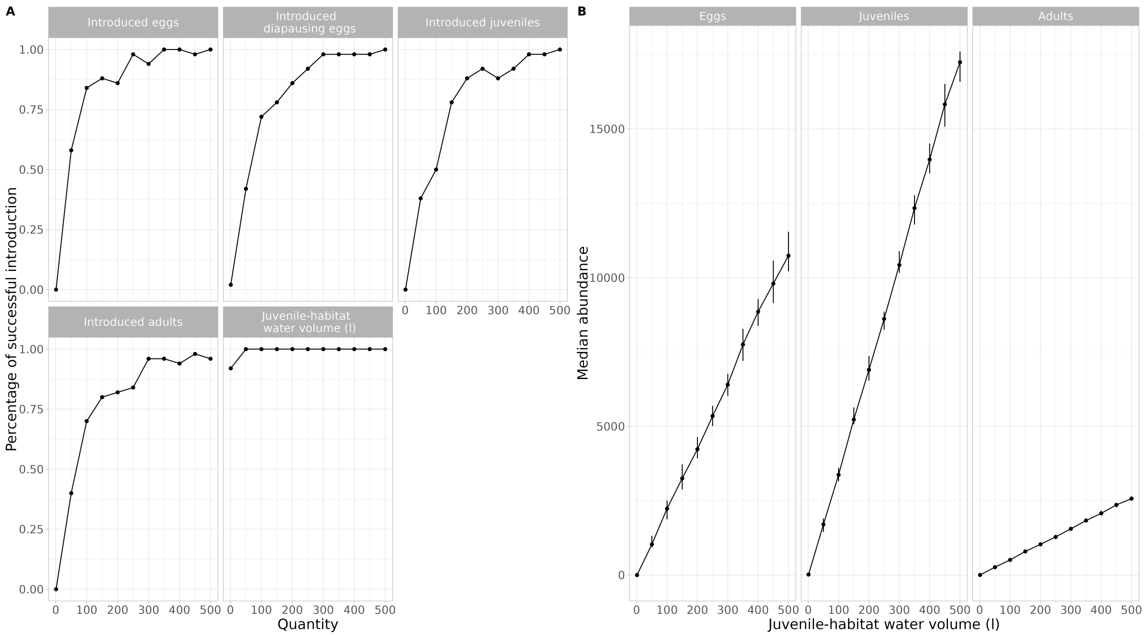

Figure 7: Sensitivity analysis on the effect of A) the variability of introduced propagules and juvenile-habitat water volume on the Percentage of successful introduction, B) the variability of the juvenile-habitat water volume on the median individual abundance.

6 *Aedes aegypti* and *Ae. albopictus* regional scale case study

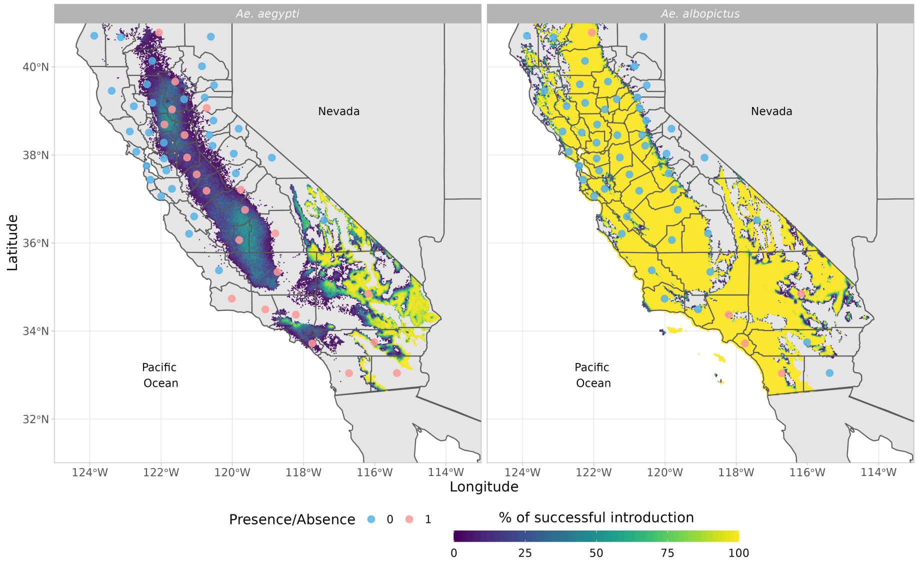

Figure 8: Predicted percentage of establishment of *Ae. aegypti Ae. albopictus* in California (USA) for the years 2011-2016 and 2013-2018, respectively. Only pixels having a probability of successful introduction >0 are shown. The red dots represent the counties where the species have been found.

7 *Aedes koreicus* population dynamics punctual scale case study

Year	Month	CI 2.5%	CI 25%	CI 50%	CI 75%	CI 97.5%	Observed *Ae. Koreicus*
2016	May	0.00	1.45	5.26	13.74	31.22	2
2016	June	0.00	1.37	6.36	14.40	40.64	5
2016	July	0.00	2.32	11.85	32.62	101.76	3
2016	August	0.70	24.57	66.65	128.03	266.00	9
2016	September	0.18	14.52	37.37	88.27	175.66	37
2016	October	0.00	0.24	1.18	3.14	10.17	0
2017	May	0.02	0.51	2.67	7.85	18.44	9
2017	June	0.29	2.75	10.60	28.57	55.28	8
2017	July	3.67	10.91	30.62	85.53	186.93	10
2017	August	7.24	32.50	74.18	159.67	283.74	24
2017	September	0.61	8.79	31.48	88.08	156.47	2
2017	October	0.08	0.82	2.20	4.98	11.06	2
2017	November	0.00	0.00	0.00	0.00	0.14	0
2018	April	1.66	5.26	11.54	22.25	34.30	0
2018	May	1.73	3.22	5.73	9.18	13.21	1
2018	June	1.57	5.81	10.52	22.29	32.89	6
2018	July	13.74	29.99	48.04	74.34	98.01	7
2018	August	37.83	71.67	109.27	157.55	201.00	29
2018	September	54.55	82.03	112.57	147.58	179.09	24
2018	October	2.13	2.90	3.77	5.89	7.80	6
2018	November	0.00	0.00	0.00	0.08	0.15	0

Table 6: Model validation for *Aedes koreicus* model in Trento (NE Italy)

References

Armistead, J., Arias, J., Nishimura, N., and Lounibos, L. (2008). Interspecific larval competition between *Aedes albopictus* and *Aedes japonicus* (Diptera: Culicidae) in Northern Virginia. *Journal of medical entomology*, 45(4):629–637.

Christophers, S. R. (1960). Aedes aegypti *(L.) The Yellow Fever Mosquito: Its Life History, Bionomics and Structure*. Cambridge University Press. Google-Books-ID: TBcf5DTKL1oC.

Da Re, D., Montecino-Latorre, D., Vanwambeke, S. O., and Marcantonio, M. (2021). Will the yellow fever mosquito colonise Europe? Assessing the re-introduction of *Aedes aegypti* using a process-based population dynamical model. *Ecological Informatics*, 61:101180.

Delatte, H., Paupy, C., Dehecq, J., Thiria, J., Failloux, A., and Fontenille, D. (2008). *Aedes albopictus* , vecteur des virus du chikungunya et de la dengue à la Réunion : biologie et contrôle. *Parasite*, 15(1):3–13.

Eisen, L., Monaghan, A. J., Lozano-Fuentes, S., Steinhoff, D. F., Hayden, M. H., and Bieringer, P. E. (2014). The impact of temperature on the bionomics of *Aedes (Stegomyia) aegypti* , with special reference to the cool geographic range margins. *Journal of medical entomology*, 51(3):496–516.

Erguler, K., Chandra, N. L., Proestos, Y., Lelieveld, J., Christophides, G. K., and Parham, P. E. (2017). A large-scale stochastic spatiotemporal model for *Aedes albopictus*-borne chikungunya epidemiology. *PloS one*, 12(3):e0174293.

Erguler, K., Smith-Unna, S. E., Waldock, J., Proestos, Y., Christophides, G. K., Lelieveld, J., and Parham, P. E. (2016). Large-scale modelling of the environmentally-driven population dynamics of temperate *Aedes albopictus* (skuse). *PloS one*, 11(2):e0149282.

Eritja, R., Palmer, J. R., Roiz, D., Sanpera-Calbet, I., and Bartumeus, F. (2017). Direct evidence of adult *Aedes albopictus* dispersal by car. *Scientific Reports*, 7(1):1–15.

Farnesi, L. C., Martins, A. J., Valle, D., and Rezende, G. L. (2009). Embryonic development of aedes aegypti (diptera: Culicidae): influence of different constant temperatures. *Memórias do Instituto Oswaldo Cruz*, 104:124–126.

Focks, D., Haile, D., Daniels, E., and Mount, G. (1993a). Dynamic life table model for *Aedes aegypti* (Diptera: Culicidae): simulation results and validation. *Journal of medical entomology*, 30(6):1018–1028.

Focks, D. A., Haile, D., Daniels, E., and Mount, G. A. (1993b). Dynamic life table model for *Aedes aegypti* (Diptera: Culicidae): analysis of the literature and model development. *Journal of medical entomology*, 30(6):1003–1017.

Grech, M. G., Sartor, P. D., Almirón, W. R., and Ludueña-Almeida, F. F. (2015). Effect of temperature on life history traits during immature development of aedes aegypti and culex quinquefasciatus (diptera: Culicidae) from córdoba city, argentina. *Acta tropica*, 146:1–6.

Hancock, P. A., Linley-White, V., Callahan, A. G., Godfray, H. C. J., Hoffmann, A. A., and Ritchie, S. A. (2016). Density-dependent population dynamics in *Aedes aegypti* slow the spread of wMel *Wolbachia*. *Journal of Applied Ecology*, 53(3):785–793.

Iwamura, T., Guzman-Holst, A., and Murray, K. A. (2020). Accelerating invasion potential of disease vector *Aedes aegypti* under climate change. *Nature Communications*, 11(1):1–10.

Iyaloo, D. P., Degenne, P., Elahee, K. B., Seen, D. L., Bheecarry, A., and Tran, A. (2021). Albomaurice: A predictive model for mapping aedes albopictus mosquito populations in mauritius. *SoftwareX*, 13:100638.

Kerkow, A., Wieland, R., Gethmann, J. M., Hölker, F., and Lentz, H. H. (2022). Linking a compartment model for west nile virus with a flight simulator for vector mosquitoes. *Ecological Modelling*, 464:109840.

Kettle, H. and Nutter, D. (2015). stagePop: modelling stage-structured populations in R. *Methods in Ecology and Evolution*, 6(12):1484–1490. _eprint: https://besjournals.onlinelibrary.wiley.com/doi/pdf/10.1111/2041-210X.12445.

Krupa, E., Henon, N., and Mathieu, B. (2021). Diapause characterisation and seasonality of *Aedes japonicus japonicus* (Diptera, Culicidae) in the Northeast of France. *Parasite*, 28.

Kurucz, K., Manica, M., Delucchi, L., Kemenesi, G., and Marini, G. (2020). Dynamics and distribution of the invasive mosquito *Aedes koreicus* in a temperate European city. *International journal of environmental research and public health*, 17(8):2728.

Lacour, G., Chanaud, L., L'Ambert, G., and Hance, T. (2015). Seasonal synchronization of diapause phases in *Aedes albopictus* (Diptera: Culicidae). *PloS one*, 10(12):e0145311.

Magori, K., Legros, M., Puente, M. E., Focks, D. A., Scott, T. W., Lloyd, A. L., and Gould, F. (2009). Skeeter Buster: A Stochastic, Spatially Explicit Modeling Tool for Studying *Aedes aegypti* Population Replacement and Population Suppression Strategies. *PLOS Neglected Tropical Diseases*, 3(9):e508.

Marcantonio, M., Reyes, T., and Barker, C. M. (2019). Quantifying *Aedes aegypti* dispersal in space and time: a modeling approach. *Ecosphere*, 10(12):e02977.

Marini, F., Caputo, B., Pombi, M., Travaglio, M., Montarsi, F., Drago, A., Rosà, R., Manica, M., and Torre, A. d. (2019a). Estimating Spatio-Temporal Dynamics of *Aedes albopictus* Dispersal to Guide Control Interventions in Case of Exotic Arboviruses in Temperate Regions. *Scientific Reports*, 9(1):1–9.

Marini, G., Arnoldi, D., Baldacchino, F., Capelli, G., Guzzetta, G., Merler, S., Montarsi, F., Rizzoli, A., and Rosà, R. (2019b). First report of the influence of temperature on the bionomics and population dynamics of *Aedes koreicus*, a new invasive alien species in Europe. *Parasites & vectors*, 12(1):1–12.

Metelmann, S., Caminade, C., Jones, A. E., Medlock, J. M., Baylis, M., and Morse, A. P. (2019). The UK's suitability for *Aedes albopictus* in current and future climates. *Journal of The Royal Society Interface*, 16(152):20180761. Publisher: Royal Society.

Mohammed, A. and Chadee, D. D. (2011). Effects of different temperature regimens on the development of aedes aegypti (l.)(diptera: Culicidae) mosquitoes. *Acta tropica*, 119(1):38–43.

Pasaoglu, G., Fiorello, D., Martino, A., Scarcella, G., Alemanno, A., Zubaryeva, A., and Thiel, C. (2012). Driving and parking patterns of European car drivers-a mobility survey. *Luxembourg: European Commission Joint Research Centre*.

Pasquali, S., Mariani, L., Calvitti, M., Moretti, R., Ponti, L., Chiari, M., Sperandio, G., and Gilioli, G. (2020). Development and calibration of a model for the potential establishment and impact of *Aedes albopictus* in Europe. *Acta tropica*, 202:105228.

Poletti, P., Messeri, G., Ajelli, M., Vallorani, R., Rizzo, C., and Merler, S. (2011). Transmission potential of chikungunya virus and control measures: the case of italy. *PloS one*, 6(5):e18860.

Reuss, F., Wieser, A., Niamir, A., Bálint, M., Kuch, U., Pfenninger, M., and Müller, R. (2018). Thermal experiments with the asian bush mosquito (*Aedes japonicus japonicus*)(Diptera: Culicidae) and implications for its distribution in Germany. *Parasites & vectors*, 11(1):1–10.

Thomas, S. M., Obermayr, U., Fischer, D., Kreyling, J., and Beierkuhnlein, C. (2012). Low-temperature threshold for egg survival of a post-diapause and non-diapause European aedine strain, *Aedes albopictus* (Diptera: Culicidae). *Parasites & Vectors*, 5(1):100.

Tran, A. and Demarchi, M. (2019). Arbocarto. modélisation spatiale dynamique des population d'aedes albopictus. france métropolitaine et régions d'outre-mer. rapport final, france métropolitaine.

Tran, A., l'Ambert, G., Lacour, G., Benoît, R., Demarchi, M., Cros, M., Cailly, P., Aubry-Kientz, M., Balenghien, T., and Ezanno, P. (2013). A rainfall-and temperature-driven abundance model for *Aedes albopictus* populations. *International journal of environmental research and public health*, 10(5):1698–1719.

Tran, A., Mangeas, M., Demarchi, M., Roux, E., Degenne, P., Haramboure, M., Le Goff, G., Damiens, D., Gouagna, L.-C., Herbreteau, V., et al. (2020). Complementarity of empirical and process-based approaches to modelling mosquito population dynamics with *Aedes albopictus* as an example—application to the development of an operational mapping tool of vector populations. *PloS one*, 15(1):e0227407.

Wieser, A., Reuss, F., Niamir, A., Müller, R., O'Hara, R. B., and Pfenninger, M. (2019). Modelling seasonal dynamics, population stability, and pest control in *Aedes japonicus japonicus* (Diptera: Culicidae). *Parasites & vectors*, 12(1):1–12.

Yang, H., Macoris, M., Galvani, K., Andrighetti, M., and Wanderley, D. (2009). Assessing the effects of temperature on the population of aedes aegypti, the vector of dengue. *Epidemiology & Infection*, 137(8):1188–1202.
